# CathAI: fully automated coronary angiography interpretation and stenosis estimation

**DOI:** 10.1038/s41746-023-00880-1

**Published:** 2023-08-11

**Authors:** Robert Avram, Jeffrey E. Olgin, Zeeshan Ahmed, Louis Verreault-Julien, Alvin Wan, Joshua Barrios, Sean Abreau, Derek Wan, Joseph E. Gonzalez, Jean-Claude Tardif, Derek Y. So, Krishan Soni, Geoffrey H. Tison

**Affiliations:** 1 Division of Cardiology, Department of Medicine, University of California, San Francisco, Cardiology, 505 Parnassus Avenue, San Francisco, CA 94143 USA; 2 Division of Cardiology, Department of Medicine, Montreal Heart Institute - Université de Montréal, 5000 Rue Belanger, Montreal, QC H1T 1C8 Canada; 3 Cardiovascular Research Institute, University of California, San Francisco, CA 94143 USA; 4 Division of Cardiology, Department of Medicine, University of Ottawa Heart Institute, University of Ottawa, 40 Ruskin Street, Ottawa, ON K1Y 4W7 Canada; 5 Department of Electrical Engineering and Computer Science, RISE Lab, University of California, Berkeley, Soda Hall, Berkeley, CA 94720-1770 USA; 6 Bakar Computational Health Sciences Institute, University of California, San Francisco, 94158 USA

**Keywords:** Acute coronary syndromes, Translational research

## Abstract

Coronary angiography is the primary procedure for diagnosis and management decisions in coronary artery disease (CAD), but ad-hoc visual assessment of angiograms has high variability. Here we report a fully automated approach to interpret angiographic coronary artery stenosis from standard coronary angiograms. Using 13,843 angiographic studies from 11,972 adult patients at University of California, San Francisco (UCSF), between April 1, 2008 and December 31, 2019, we train neural networks to accomplish four sequential necessary tasks for automatic coronary artery stenosis localization and estimation. Algorithms are internally validated against criterion-standard labels for each task in hold-out test datasets. Algorithms are then externally validated in real-world angiograms from the University of Ottawa Heart Institute (UOHI) and also retrained using quantitative coronary angiography (QCA) data from the Montreal Heart Institute (MHI) core lab. The CathAI system achieves state-of-the-art performance across all tasks on unselected, real-world angiograms. Positive predictive value, sensitivity and F1 score are all ≥90% to identify projection angle and ≥93% for left/right coronary artery angiogram detection. To predict obstructive CAD stenosis (≥70%), CathAI exhibits an AUC of 0.862 (95% CI: 0.843–0.880). In UOHI external validation, CathAI achieves AUC 0.869 (95% CI: 0.830–0.907) to predict obstructive CAD. In the MHI QCA dataset, CathAI achieves an AUC of 0.775 (95%. CI: 0.594–0.955) after retraining. In conclusion, multiple purpose-built neural networks can function in sequence to accomplish automated analysis of real-world angiograms, which could increase standardization and reproducibility in angiographic coronary stenosis assessment.

## Introduction

Coronary heart disease (CHD) is the leading cause of adult death in the United States and worldwide^1^, caused by atherosclerotic plaques narrowing the coronary arteries, also called coronary artery disease (CAD). The coronary angiography procedure is the gold standard procedure to diagnose coronary artery stenosis which therefore provides crucial information for CAD treatment decisions ranging from medical therapy alone to revascularization with coronary stents or bypass surgery^2^. Physician visual estimation of coronary stenosis severity from angiograms remains the most common, guideline-supported approach to evaluate angiographic narrowing of the coronary artery lumen^2,3^.

However, the limitations of visual estimation for coronary stenosis severity are well described, and include intra- and inter-observer variability, operator bias and poor reproducibility^4,5^. Variability in visual stenosis assessment ranges from 7.6 to 22.5%^4–6^. And while coronary angiography imaging quality has improved, variability still remains and is greater in the setting of a single physician reader, which is by far the most common clinical workflow^4,7^. Visual assessment of stenosis can overestimate the severity of stenosis in over a quarter of cases^8^ and may contribute to inappropriate coronary artery bypass surgery in 17% of patients and stent usage in at least 10% of patients^4–6,8^. A more standardized, reproducible approach to angiogram interpretation and coronary stenosis assessment would have substantial clinical importance.

Various adjunctive testing may supplement CAD assessment during coronary angiography, such as physiologic assessment^9,10^ or intra-vascular imaging^11^, though most require additional operator expertise and use of additional catheters, thus are used in less than 10–20% of coronary angiograms^9,11,12^. Furthermore, determining the need for adjunctive testing still relies primarily upon physician visual estimation of angiograms during the angiography procedure to identify intermediate-severity or greater coronary stenosis (e.g. 40–69%)^3^. Quantitative coronary angiography (QCA) is a technique providing analysis of angiograms that allows for more standardized stenoses assessment^13^. However, QCA is not fully automated and requires operator input for image calibration and frame selection, relegating it largely to research settings^5,14^.

Artificial intelligence (AI) algorithms, and deep neural networks in particular, have demonstrated the ability to automate important clinical tasks in cardiology as well as interventional cardiology^15^ Our objective is to develop and validate an automated approach for coronary angiogram interpretation, coronary artery stenosis localization and severity estimation from real-world coronary angiograms.

## Results

**Fig. 1:**
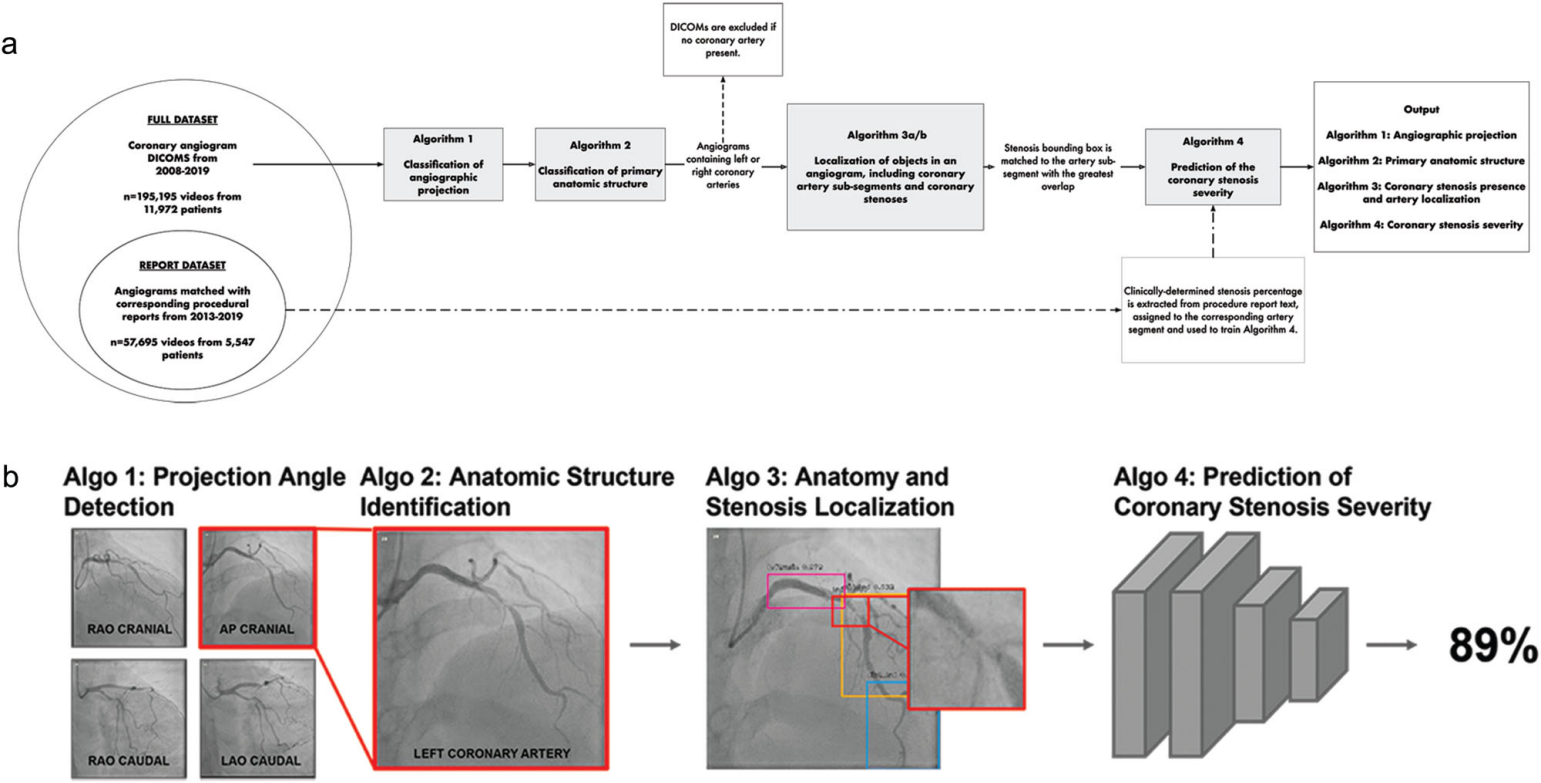
Overview of CathAI. **a** Overview of the CathAI pipeline for automated angiogram interpretation. Angiogram videos flow from one algorithm to the next to accomplish the 4 critical tasks required for automated interpretation. **b** Application of CathAI to an example coronary angiogram. An example left anterior descending artery angiogram with severe stenosis (proximal to mid segment) is shown progressing through the CathAI system to: identify angiographic projection, identify LCA (left), place bounding boxes around objects such as stenosis (zoom, middle), and predict maximal stenosis severity (right).

The Full Dataset consisted of 13,843 complete angiogram studies (195,195 total angiographic videos) from 11,972 patients aged ≥18 years from the University of California, San Francisco (UCSF), between April 1, 2008 and December 31, 2019 (Fig. 1a, Supplementary Fig. 1). Mean age was 63.5 ± 13.7 years in the Full Dataset and 66.7 ± 12.0 in the Report Dataset.

### CathAI performance to classify angiographic projection angle, anatomic structures, and angiogram object localization

To classify angiographic projection angle, CathAI achieved a frequency-weighted positive predictive value (PPV) of 0.90, sensitivity of 0.90 and F1 score of 0.90 in the test dataset (Supplementary Table 8). Highly used projection angles, such as LAO Straight, RAO Straight and LAO Cranial, had better overall performance. To classify the primary anatomic structure contained in an angiogram video, CathAI achieved a frequency-weighted PPV of 0.89, sensitivity of 0.89 and F1 score of 0.89 in the test dataset (Table 1). CathAI performance was high for both the left and right coronary arteries (LCA and RCA, respectively), which are the primary angiographic objects of interest: PPV and sensitivity, respectively, were 0.97 and 0.94 for LCA and 0.93 and 0.93 for RCA.

**Table 1:** CathAI classification of the Anatomic Structure at the frame-level in the test dataset (Algorithm 2).

Anatomic class	Positive predictive value	Sensitivity	F1 score	Number of images	Number of unique videos
Left coronary artery	0.97	0.94	0.95	1055	534
Right coronary artery	0.93	0.93	0.93	632	254
Bypass graft	0.49	0.62	0.62	71	15
Percutaneous coronary intervention	0.85	0.79	0.82	290	114
Catheter	0.78	0.91	0.84	512	236
Pigtail Catheter	0.69	0.55	0.55	44	32
Ventriculography	1.00	0.67	0.67	15	2
Radial Artery	0.55	0.63	0.63	11	3
Femoral Artery	0.95	0.97	0.97	286	136
Aortography	0.75	0.75	0.75	4	1
Other	0.44	0.44	0.44	55	26
Frequency- weighted average	0.89	0.89	0.89	2975	1353

Results are calculated on the hold-out Test Dataset for Algorithm 2.

Once angiogram videos primarily containing the LCA and RCA were identified, CathAI localized objects in the angiogram by predicting bounding boxes around coronary artery segments such as the proximal portion of a coronary artery, stenosis regions, stents and coronary catheters. To measure CathAI performance to localize these objects, mean average precision (mAP) was used to compare predicted coordinates against ground-truth for each object. CathAI exhibited a 48.1% weighted average mAP in the test dataset, which corresponds to state-of-the-art results for object-localization AI algorithms^16^ (Supplementary Table 9). CathAI correctly localized 93.3% of coronary artery stenoses (PPV) in UCSF test dataset.

### CathAI performance to predict stenosis severity

**Fig. 2:**
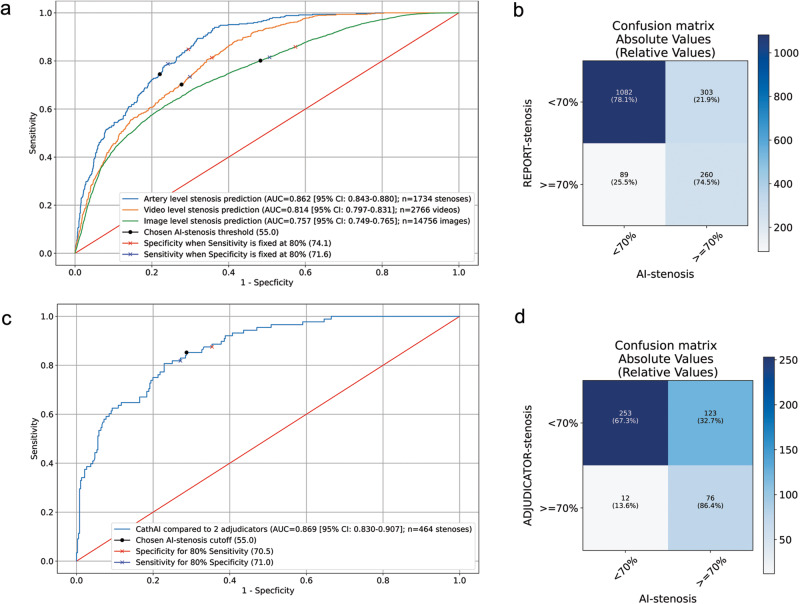
CathAI performance to predict obstructive coronary artery stenosis. **a** Receiver Operating Characteristic Curves (ROC) for CathAI prediction of obstructive (</≥70%) coronary stenosis in the test dataset. Black dot: AI-stenosis threshold chosen to optimize F1 score in the artery-level dataset. Red cross: Specificity when sensitivity is fixed at 80%. Blue cross: Sensitivity when specificity is fixed at 80%. **b** Confusion matrix for CathAI prediction of obstructive stenosis vs. REPORT-stenosis. **c** UOHI External Validation Dataset-ROC for CathAI prediction of obstructive (</≥70%) stenosis. Black dot: AI-stenosis threshold chosen to optimize F1 score in the artery-level dataset. Red cross: Specificity when sensitivity is fixed at 80%. Blue cross: Sensitivity when specificity is fixed at 80%. **d** UOHI External Validation Dataset-Confusion matrix for CathAI prediction of obstructive stenosis vs. expert adjudicators. AUC area under the curve, CI confidence interval, AI artificial intelligence.

The final algorithm in the CathAI system estimated coronary artery stenosis severity (Fig. 1b). Predicted estimates of stenosis severity were averaged from all angiogram videos from a given study that visualized a particular artery segment (called “artery-level”), mirroring standard clinical practice. In the test dataset, CathAI’s AUC to identify obstructive stenosis was 0.862 (95% CI: 0.843–0.880) at the artery-level (Table 2; Fig. 2a). Artery-level stenosis prediction performed better than video or image level predictions (Fig. 2a, Supplementary Table 10). For CathAI’s prediction of stenosis severity as a continuous percentage stenosis, the mean absolute percentage difference between the AI-stenosis and report-stenosis was 17.9 ± 15.5% (Table 2; Supplementary Fig. 3). There was a significantly lower mean absolute percentage difference for the RCA versus the LCA (16.4 ± 15.0 vs 19.0 ± 15.8; *p* < 0.001, Table 2; Supplementary Table 10), at similar training dataset sizes—likely reflecting the RCA having less anatomic variation than the left. CathAI had modestly higher AUC to identify severe stenosis in strata of females vs males [0.890 (95% CI: 0.864–0.923) vs 0.830 (95% CI: 0.805–0.856) respectively, *P* value for interaction: 0.02].

**Table 2:** Performance of AI-stenosis (CathAI) versus REPORT-stenosis in the test dataset.

	Number of REPORT-stenosis labels	AUC (95% CI) to discriminate </≥ 70% stenosis severity	Mean Absolute difference, % stenosis (continuous) ± SD	r* (95% CI)	ICC* (95% CI)	Sensitivity (95% CI)**	Specificity (95% CI)** for </≥ 70% stenosis severity	PPV (95% CI)** for </≥ 70% stenosis severity	NPV (95% CI)** for </≥ 70% stenosis severity	*P* value for Interaction
Overall (Artery level)	1734	0.862 (0.843–0.880)	17.9 ± 15.5	0.74 (0.72–0.76)	0.72 (0.60–0.84)	74.5 (70.0–78.4)	78.1 (76.1–80.1)	46.1 (42.2–50.2)	92.4 (90.9–93.6)	–
Left Coronary Artery	980	0.835 (0.808–0.862)	19.0 ± 15.8	0.72 (0.68–0.74)	0.69 (0.54–0.85)	76.1 (71.0–81.3)	72.2 (68.9–75.3)	42.6 (38.0–47.1)	93.3 (91.8–95.0)	Ref
Right Coronary Artery	754	0.894 (0.869–0.919)	16.4 ± 15.0	0.77 (0.74–0.80)	0.75 (0.65–0.87)	70.9 (62.7–78.0)	85.6 (83.2–87.9)	50.1 (43.0–57.1)	82.7 (80.1–85.6)	0.11
Sex***										
Male	1058	0.830 (0.805–0.856)	18.5 ± 15.3	0.73 (0.70–0.75)	0.68 (0.55–0.83)	78.6 (73.8–82.9)	70.6 (68.1–73.3)	47.7 (43.5–52.5)	90.6 (88.2–92.5)	Ref
Female	672	0.890 (0.864–0.923)	16.9 ± 15.8	0.75 (0.63–0.87)	0.74 (0.70–0.77)	80.2 (72.0–88.4)	78.9 (75.6–82.0)	35.6 (29.2–40.2)	96.6 (95.2–97.9)	**0.02**

*ICC* intra-class correlation, *r* Pearson correlation, *AUC* area under the receiver operating characteristic curve, *PPV* positive predictive value, *NPV* negative predictive value, *MSE* mean squared error.

I*Calculation is between AI-stenosis (continuous) and REPORT-stenosis; **The threshold for determining sensitivity, specificity, PPV and NPV for AI-stenosis was 54%. The interaction *P* values were calculated using two-sided Wald tests between the CathAI Algorithm 4 probability and the respective covariates for stenosis percentage. *P* values < 0.05 are bolded. ***Sex for *n* = 4 patients was unknown.

Of those ≥70% stenoses according to the REPORT-stenosis, CathAI classified 74.5% correctly (95% CI: 70.0–78.4%; 260/349, Fig. 2b); and of those <70% stenoses by REPORT-stenosis, Algorithm 4 classified 78.1% correctly (95% CI:76.1–80.1%; 1082/1385). When Algorithm 4’s sensitivity to detect obstructive coronary stenosis was fixed at 80.0%, its specificity to detect obstructive stenosis was 74.1%; and when specificity was fixed at 80.0%, its sensitivity to detect obstructive stenosis was 71.6%. There were medium-strong correlations between the continuous AI-stenosis and REPORT-stenoses (Table 2). CathAI overestimated milder stenoses and underestimated severe stenoses (Supplementary Fig. 3).

### External validation of CathAI in the UOHI dataset

To examine external generalizability, we applied CathAI to 464 randomly sampled angiogram videos from UOHI. CathAI predicted angiographic projection with high accuracy (Supplementary Fig. 4). In 100% of UOHI angiograms, Algorithm 2 successfully identified the RCA or LCA. The two UOHI adjudicators agreed on stenosis localization within the same coronary artery segment in 91.4% (*n* = 424). Within this subset CathAI localized stenosis in the same artery segment in 84.5% (*n* = 360); in all the remaining 15.5% where the artery sub-segment was not correct, CathAI assigned stenosis to the correct coronary artery overall (LCA vs RCA). All CathAI-identified stenoses were true stenoses, as opposed to artifact due to suboptimal opacification or vessel tortuosity, according to both adjudicators.

**Fig. 3:**
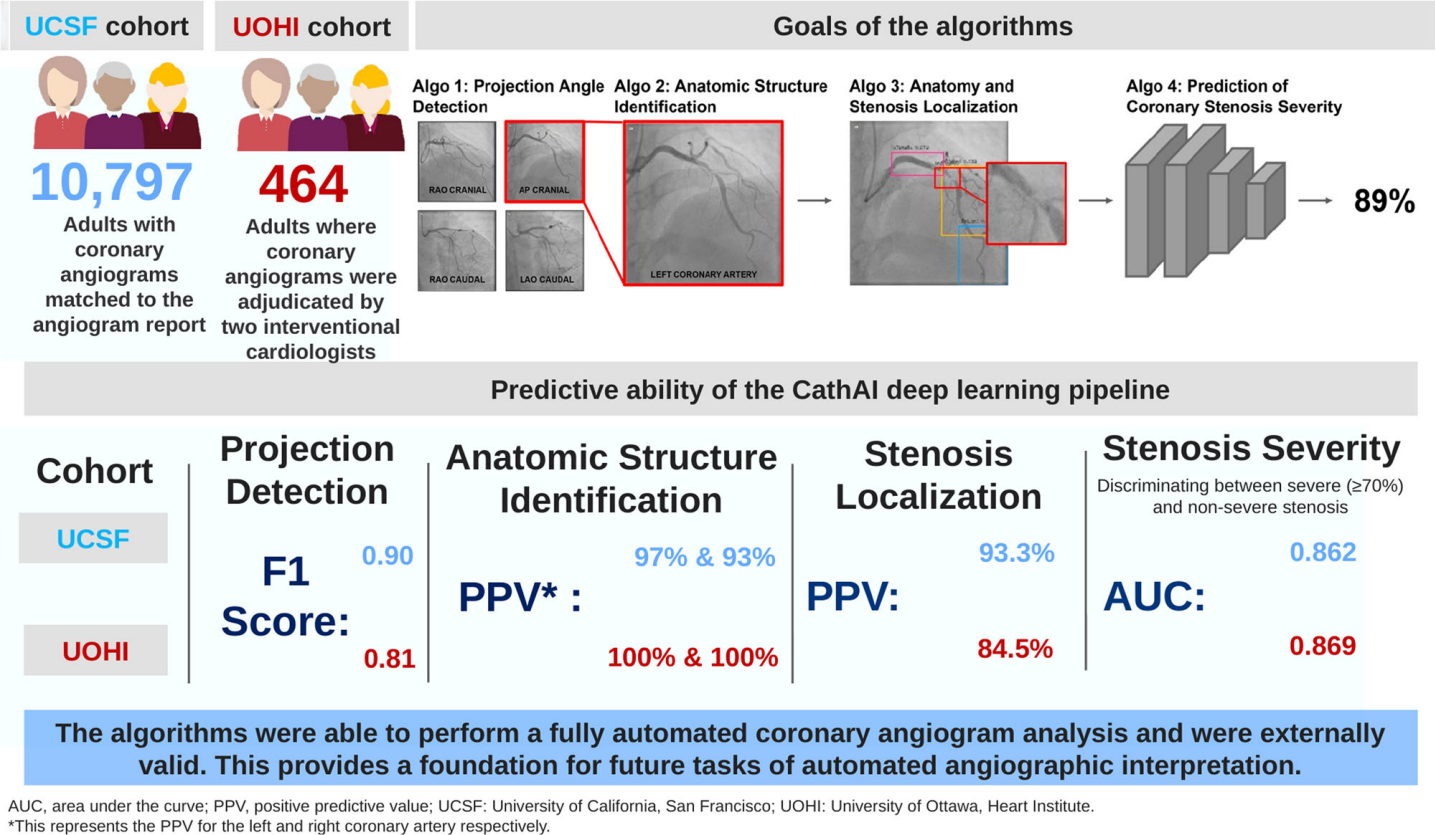
Summary of study results from CathAI internal and external validation. Main results for University of California, San Francisco (UCSF) internal validation and University of Ottawa Heart Institute (UOHI) external validation are shown for each of the 4 tasks.

Inter-observer variability for stenosis percentage assessment between the two adjudicators was 15.7% ± 14.5%. For determination of obstructive (</≥70%) stenosis, adjudicators disagreed on 16.8% of stenoses as being obstructive (*n* = 78). We calculated the arithmetic mean of the percent stenosis from the two adjudicators to compare against CathAI’s prediction of stenosis severity prediction. Compared to this, CathAI’s AUC for obstructive stenosis (≥70%) was 0.869 (95% CI: 0.830–0.907; Figs. 2c, 3); sensitivity was 86.4% and specificity was 67.3% (Fig. 2d; same threshold used as UCSF dataset, 0.54). The mean absolute percentage difference between AI-stenosis and the percent stenosis averaged from the two adjudicators was 18.02% ± 11.02%. In the UOHI dataset, CathAI took ~3–5 s to analyze an angiography video, or 35 s for a full exam, using a Nvidia GTX 1080 Ti GPU.

### Retraining CathAI to predict QCA Stenosis

The RCT QCA dataset consisted of 709 patients with 858 exams (1384 stenoses; 18 severe ≥70%, 71 severe ≥50% but <70% and 1295 non-severe). CathAI successfully identified 100% of LCA/RCA videos and 78.7% (*n* = 1384) of stenoses (67.2% for LCA and 86.8% for RCA). Average stenosis severity as assessed by QCA was 31.7 ± 11.6% (Supplementary Fig. 5). Given the divergent patient population of the RCT QCA dataset of mostly mild CAD and QCA adjudication criteria (≥50% QCA stenosis) instead of visual estimation, the RCT QCA dataset provided an opportunity to retrain CathAI to predict QCA labeled stenoses, as opposed to visually estimated stenoses. Once re-trained, CathAI’s AUC to discriminate obstructive QCA stenosis (≥50%) was 0.775 (95% CI: 0.594–0.955) at artery-level (Supplementary Table 11) and for QCA stenosis ≥70%, the AUC was 0.75 (95% CI: 0.570-0.930). The average stenosis difference between CathAI-predicted stenosis and QCA-estimated stenosis was 6.5 ± 5.5% in the test dataset.

### Using neural network explainability to understand CathAI performance

**Fig. 4:**
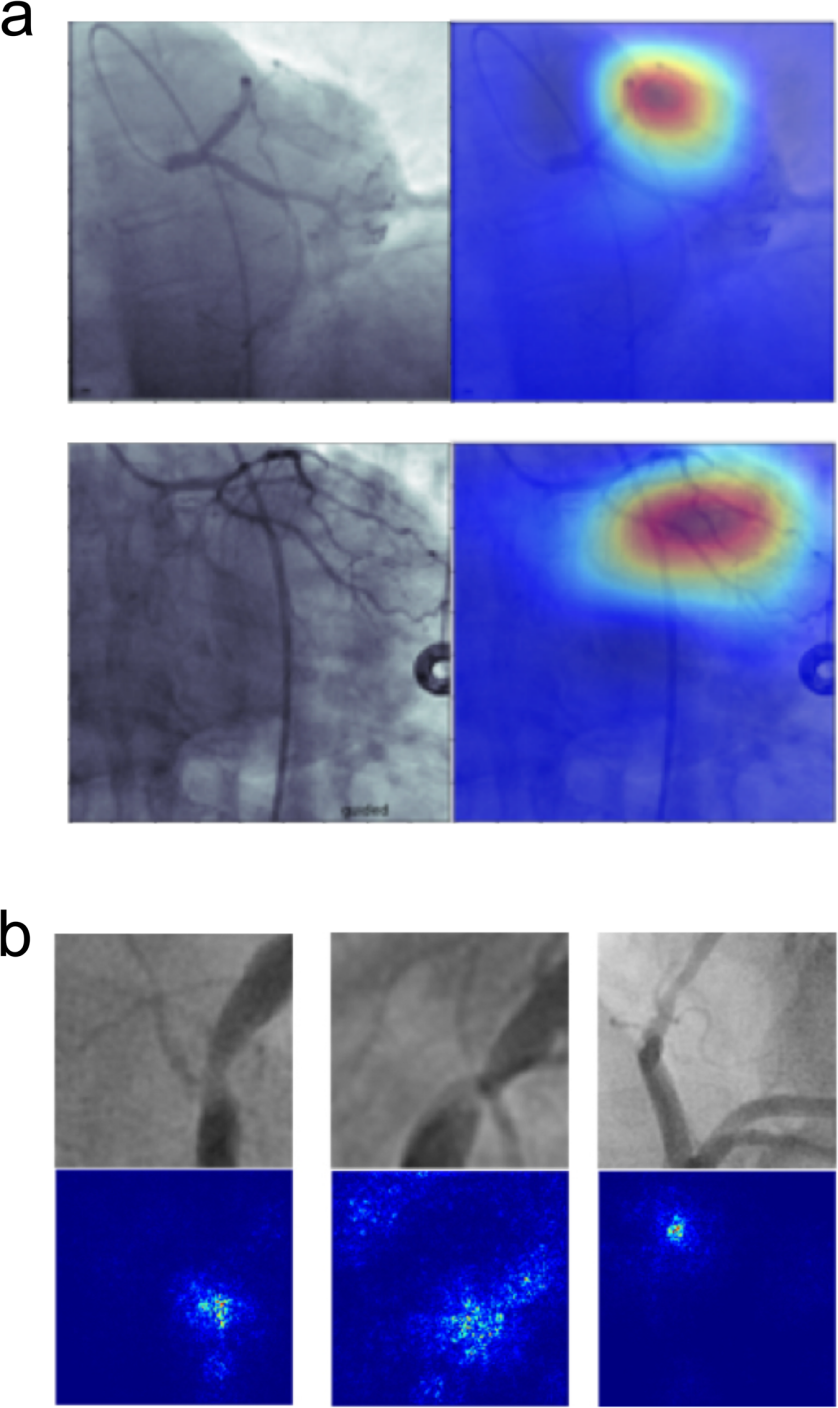
Explainability methods applied to CathAI algorithms. **a** GradCAM applied to CathAI classification of primary anatomic structure. Two original angiogram images are shown (left), alongside corresponding images highlighted by GradCAM (right) showing areas of greater importance for algorithm decisions. GradCAM-highlighted areas focused around the left coronary artery within the angiogram image, with blue color indicating lowest importance, yellow color indicating medium importance and red color indicating highest importance to CathAI Algorithm 2’s prediction. **b** LOVI Saliency Maps of CathAI prediction of coronary stenosis severity. Original angiogram images (top) and corresponding images with LOVI saliency maps (bottom). White pixels represent greater contribution to CathAI’s (Algorithm 4) prediction, showing that Algorithm 4 focused on pixels near the region of coronary artery stenosis in most cases. LOVI Layer Ordered Visualization of Information.

To better understand the elements within angiograms that contributed to CathAI predictions, we applied two explainability approaches to the fully-trained CathAI algorithms. This can help illuminate how algorithms function and provide additional decision-making context to clinicians. GradCAM^17^ highlights image regions most critical to CathAI’s prediction, showing that it used regions like a cardiologist, such as the left anterior descending artery to identify LCA images (Fig. 4a). We also derived saliency maps for CathAI’s prediction of stenosis severity using the Layer Ordered Visualization of Information (LOVI) method^18^. The highlighted pixels (Fig. 4b; Supplementary Fig. 6) were not only limited to stenosed artery segments, but also to normal segments and pixels immediately adjacent to the artery, suggesting that the relationship between stenosed and non-stenosed artery segments contributes to CathAI’s prediction of stenosis severity.

## Discussion

We described the development and validation of the CathAI pipeline comprised of four algorithms and demonstrated significant advancements in automated analysis of coronary angiograms. Each algorithm achieved a specific task that facilitated accomplishment of the primary diagnostic aim of coronary angiography—assessment of coronary artery stenosis severity—achieving state-of-the-art performance for each task. Importantly, CathAI was generalizable, without additional training, to predict visually estimated stenoses from non-curated real-world UOHI angiograms, a separate medical system in a different country. CathAI was also successfully re-trained to predict QCA stenosis estimates in a third external RCT QCA dataset. This provides a broad foundation for various future angiogram-relevant tasks—such as automatic estimation of a stenosis severity or identification of intermediate-grade stenoses requiring adjunctive testing. Furthermore, the adoption of explainability methods such as GradCAM and LOVI provides clinicians better understanding of the CathAI’s predictions.

Prior work has reported neural networks performing individual tasks in experimental settings related to angiogram analysis such as frame extraction^19^, stenosis prediction from (manually-selected) RCA images^19–25^, coronary vessel segmentation^26,27^ or stenosis identification^28^. However, to our knowledge fully automated analysis and stenosis prediction for coronary angiogram videos has not yet been demonstrated on real-world angiograms. Many experimental prior approaches focused on the RCA^19–25^ likely because it has substantially less anatomic variability and complexity compared to the LCA, decreasing the difficulty of analyzing RCA angiograms. However, approaches trained on pre-selected RCA angiograms cannot analyze real-world datasets, which include non-RCA and non-coronary artery videos. In a recent effort Du et al. analyzed single frames from both LCA and RCA angiograms to localize coronary segments and stenosis^28^, but did not predict severity of stenosis which is a critical component. Our work advances the state-of-the-art that is generalizable to real-world external angiograms and mirroring the standard-of-care guideline-recommended “worst view” assessment^2,3^. Because coronary angiography is critical to all CHD clinical decision-making^2,3^ CathAI has substantial potential to automate angiogram interpretation. The deviation of CathAI’s predictions from human experts’ visual assessment was well within, and often less than, commonly reported inter-observer variability^4–7,29^; whereas CathAI’s retrained QCA deviation was 6.5 ± 5.5% in the RCT QCA dataset which is substantially lower than the 10.2–16.6% difference between physician visual assessment and QCA reported in the literature^5^

Automated analysis of angiograms has greater similarities to the perception-side of “self-driving car” technology than to standard radiologic analysis (i.e. X-rays), given angiograms’ highly variable, operator-determined video acquisitions and the sequence of complex tasks required for analysis. Successful analysis of real-world angiograms requires the AI-system to process any type of video encountered during real-world procedures, identify relevant images, then accurately localize important objects. CathAI’s state-of-the-art performance on each core task and also stenosis estimation^19–25,28^ provides proof-of-concept that multiple purpose-built deep learning algorithms can overcome the barriers that to date have prevented fully automated angiogram analysis. The multiple sub-tasks CathAI accomplishes en route to the final stenosis prediction provides a robust foundation to support many future additional tasks that build upon any CathAI sub-task, such as predicting stent under-expansion, atherosclerotic plaque morphology or fractional flow reserve from contrast flow patterns. CathAI can be readily adapted to additional tasks. Like self-driving car technology, however, much work remains to achieve very high accuracy before CathAI is “clinically ready,” given the central role of coronary stenosis assessment to CHD clinical decision making^2,3^. Undoubtedly, additional improvement in each of CathAI’s individual algorithms will likely be needed prior to clinical deployment, achievable for example by increasing the sizes of human-expert annotated datasets or training purpose-built algorithms for specific views (such an RCA-LAO only artery localization algorithm). This work provides the foundation for rapid improvement or to develop future algorithms for additional tasks, like demonstrated for QCA retraining.

The most immediate clinical implication of deploying a pipeline such as CathAI would be to increase standardization in the assessment of coronary stenosis. Human expert visual estimation of coronary stenosis is well established to have high variability of between 7–22%^4–7,29^ and between 10–17% against a QCA gold-standard^5^, directly impacting decisions for life-saving CHD revascularization therapies. Multiple studies have consistently reported overestimation of stenosis by visual estimation^5,6,8^. One study re-evaluated clinical angiograms by multiple readers, reporting that the recommendation for coronary bypass surgery changed from “necessary/appropriate” to “uncertain/inappropriate” in 17–33% of cases, including 10% of cases for stent placements^4^. This study^4^ suggested performing a second independent angiogram interpretation before revascularization, though this is generally infeasible and not the clinical standard-of-care given clinical interventional cardiology workflows. However, CathAI could easily perform this function in an automated, reproducible manner to provide near real-time predictions during the procedure to supplement physicians’ own ad-hoc diagnosis. However, since CathAI was trained with the same biased visually estimated stenoses, it also tends to overestimate stenoses. To address this, CathAI could be re-trained using QCA data, which, as we demonstrate, tends to predict less severe stenoses as is shown in the literature.

Some recent studies have suggested that there may be sex differences in stenosis estimations, where stenoses tend to be overestimated in females^30^. To investigate whether CathAI could help reduce this bias in stenosis estimation across sex, we compared the performance of Algorithm 4 between males and females in our internal dataset. Our analysis found a small but significant difference between the algorithm’s performance in strata of males and females. Future work should aim to balance examples of severe and non-severe stenoses in both males and females to further address the issue of sex-based bias in stenosis severity estimation.

The UOHI dataset provided external validation showing that CathAI generalizes well to external real-world angiograms adjudicated by physician visual estimation. In comparison, the RCT QCA dataset represented a not only a different method of stenosis adjudication (that also uses different thresholds for severe stenosis ≥50% for QCA vs. ≥70% for visual estimation), making it effectively a different task, but a very different patient population with predominantly mild-CAD due to the RCTs’ dataset design. In most UCSF clinical angiograms, as is common in clinical practice, coronary stenoses ≤50% were simply described as “non-obstructive stenosis” and not given a percentage estimate; in contrast, QCA provides stenoses values in all cases. This is exemplified by CathAI identifying 78.7% QCA dataset stenoses compared to 100% of those in the real-world clinical UOHI datset, since the QCA dataset had predominantly mild stenosis. For these reasons, the RCT QCA dataset provided an opportunity to examine how CathAI (Algorithm 4) could provide a pre-trained foundation to learn the new task of QCA prediction using a small dataset of <500 patients with a different CAD distribution. Once retrained, CathAI’s performance to discriminate obstructive stenosis by QCA was numerically lower (AUC = 0.775) than for obstructive stenosis by visual estimation in the main analysis, possibly due in part to the small size of the QCA dataset, although confidence intervals overlapped. However, it is notable that the deviation of 6.5 ± 5.5% of CathAI’s prediction from QCA was substantially lower than the previously reported human visual estimate deviation from QCA in the PROMISE trial of 10–17%. This underscores one of the most immediate potential contributions of AI-automated analysis workflows to decrease interpreter-variability and increase standardization. We believe that this first demonstration of a retrainable CathAI automated angiogram analysis system provides a path forward for future research, highlighting the areas necessary to improve to ultimately build a clinically ready automated angiographic analysis system.

Our work has several limitations. A notable limitation arises from our use of training labels derived during routine clinical care using physician visual estimation. Due to the resource-intensive nature of generating large numbers of cardiologist annotations for angiographic images, to achieve the largest dataset to train CathAI for stenosis severity, we used clinically-generated REPORT-stenosis values which were available in large numbers. Though these were generated by sub-specialty trained interventional cardiologists at UCSF, they likely still exhibit variability inherent in any clinically generated label. Such variability in both the training and testing data could place an artificial ceiling on observed algorithmic performance; however these CathAI algorithms can be easily re-trained and refined with purpose-generated labels. To demonstrate this, we retrained CathAI with QCA stenosis labels, whose performance could likely be improved with a larger QCA dataset. For example, our QCA dataset had very low number of severe stenoses (≥70%) therefore future efforts should increase examples of severe stenoses adjudicated by QCA. In addition, the text-parsing method we used to extract the REPORT-stenosis from the clinical procedure report may have introduced errors in either the location of the stenosis or the degree of severity. Future algorithm improvements will likely come from using purpose-generated labels, such as from a core-lab using either protocol-guided visual assessment, QCA, or physiologic assessment such as FFR^31^. Our objective with this proof-of-concept study was to demonstrate the “building blocks” required for automatic interpretation of coronary angiograms, and not necessarily to replace the current clinical standard for angiogram interpretation. To achieve the latter, substantial human effort will likely be required to label large datasets with precise methods. Furthermore, we only included the main epicardial vessels in this version of CathAI. Other pertinent vessels/objects, like bypass grafts, diagonals, chronic total occlusions or collaterals were excluded, but represent prime targets for near-term future work.

In conclusion, CathAI is the first multi-stage fully automated analysis pipeline for coronary angiograms. CathAI achieves state-of-the-art performance for each task required for interpretation of real-world angiograms, is externally valid, and provides a foundation for future tasks in automated angiographic interpretation. The automated stenosis assessment enabled by CathAI may serve to increase standardization and reproducibility in coronary stenosis assessment, one of the most critical junctures in CHD clinical decision making.

## Methods

### Study participants and study datasets

For our Full Dataset, we obtained retrospective, de-identified coronary angiographic studies from all patients 18 years or greater from the University of California, San Francisco (UCSF), between April 1, 2008 and December 31, 2019 (Supplementary Fig. 1) that underwent a coronary angiogram. Patients without videos of the left or right coronary artery were excluded. Angiograms were acquired with Philips (Koninklijke Philips N.V., Amsterdam, Netherlands) and Siemens (Siemens Healthineers, Forchheim, Germany) systems at 15 frames per second using Iopromide contrast. We generated specifically annotated training datasets (either through available meta-data or expert annotation) of Full Dataset subsets for each of the four primary tasks performed by CathAI.

To maximize the manual labeling efforts required to generate training data for each Algorithm, we generated an “extracted Full dataset” by first automatically identifying the frames within the video that likely contained peak-contrast by calculating the structural similarity index measure (SSIM) from the frame in position ‘0’ where no dye is usually present, which we called the “reference” frame. SSIM is higher if images have similar pixel values and lower if there is greater difference. The frame with the lowest similarity index from the reference frame was selected as most likely containing peak-contrast (e.g. when the artery is full of contrast). Up to 8 frames were then extracted from each video by retaining the reference frame, the peak-contrast frame and the 3 frames immediately preceding and following the peak-contrast frame. This is referred to as the “extracted” Full Dataset frames (*n* = 1,418,297). All frames of a video were converted to images of dimension 512*512 pixels for algorithmic analysis. Subsets of frames from the extracted Full Dataset were then labeled for each task, as described below, to generate training data for Algorithms 1–3.

For all algorithms, except Algorithm 3, data was split randomly for each algorithm into Training (70%), Development (10%) and Test (20%) datasets, each containing non-overlapping patients. The development dataset was used for algorithm tuning, when required. For Algorithm 3, dataset splits were Training (80%) and Test (20%); since we used original hyperparameters and did not require algorithm tuning^16,32^.

Algorithm 1 labels were taken directly from the DICOM metadata describing the cranial-caudal and LAO-RAO orientations. Algorithm 2 and 3 required annotations by a board-certified cardiologist (Supplementary Fig. 8 and Supplementary Table 1, 2 and 3 for definitions). For Algorithm 4, the stenoses were taken directly from the procedural report.

### The Report dataset

Shortly after performing the procedure, interventional cardiologists typically interpret the angiogram using visual assessment, as per standard clinical practice, and describe the severity of coronary stenosis in the procedure report. This procedural report text was parsed (see below) to identify: any description of coronary artery stenosis, the maximal stenosis percentage (called the REPORT-stenosis) and its location in one of 11 coronary artery segments (Supplementary Table 3). We identified 9782 coronary stenoses in artery segments (REPORT-stenoses) and identified in 1766 non-stenosed complete vessels yielding a total of 10,088 non-stenosed vessel segments, derived from 84,153 images. Then we randomly sampled 10,000 images corresponding to healthy artery subsegments (Supplementary Fig. 1). Metadata was extracted from each angiogram video including the procedure date, the primary (Right Anterior Oblique [RAO]/Left Anterior Oblique [LAO]) and secondary (cranio-caudal) angles of rotation, a unique exam identifier and a unique patient identifier. Non-matched REPORT-stenoses were removed from the dataset. We also excluded videos where an intra-coronary guidewire was present in more than 4 frames, as automatically determined by Algorithm 3 (6,076 videos, 41,780 stenosis-frames identified with guidewires), since these videos likely represent percutaneous coronary interventions which could alter the stenosis percentage within that video (Supplementary Fig. 1); videos were retained from studies prior to the insertion of an intracoronary guidewire.

### Text parsing methods

The free text from the procedural report was first segmented using commas (“,”) or periods (“.”). We then applied text parsing methods to identify distinct coronary segments (Supplementary Table 3). When a coronary segment was found, we identified any description of corresponding stenosis percentage by localizing “%” and the nearest one to three-digit number in that sentence. We initially searched using standard terms (such as “right coronary artery”), then expanded the keywords by manual review of the text, over multiple iterations, to include the most common abbreviations, alternate spellings and permutations (Such as “RCA”). Qualitative descriptions of obstructive CHD, such as “mild”, “moderate” or “severe” disease were not extracted. Furthermore, we searched for keywords such as “thrombus”, “obstruction” or “occlusion” in the report; when present in a coronary segment, 100% stenosis was assigned to that segment. For analysis, ostial and proximal coronary segments were merged in the ‘proximal’ class and ostial, proximal, middle, and distal left main arteries were merged under the ‘left main’ class. The most severe stenosis within any of the 11 segments was retained (Supplementary Table 3). We did not analyze chronic total occlusions and stenoses in diagonals, marginals, septals, ramus, left posterior descending artery, left posterolateral or in bypass grafts.

### Human subjects research

This study was reviewed by the University of California, San Francisco Institutional Review Board and need for informed consent was waived. The external validation was reviewed and approved by the University of Ottawa Institutional Review Board.

### Algorithm development

The CathAI system is comprised of 4 neural network algorithms organized in a pipeline (Fig. 1a). Angiographic images are analyzed by each algorithm and “flow” sequentially to the next to accomplish the four foundational tasks for automated angiogram analysis: (1) classification of angiographic projection angle; (2) classification of an angiogram’s primary anatomic structure; (3) localization of relevant objects within an angiogram, including coronary artery sub-segments and stenoses; (4) prediction of coronary artery stenosis severity (as a percentage of artery narrowing).

We customized each of CathAI’s 4 algorithms base neural network architecture to achieve an angiogram-relevant task, as detailed in sections below. As a high-level summary, Algorithm 1 accepted individual images (coronary angiogram video frames) as input and identified the angiographic projection angle used described by LAO-RAO and cranial-caudal axes (LAO cranial, RAO caudal, etc); labels were available from each video’s metadata. Algorithm 2 identified the primary anatomic structure (Supplementary Table 2), since it is common to capture angiogram videos containing non-cardiac anatomic structures such as the aorta or the femoral artery. Algorithm 2 allowed CathAI to subsequently focus on only angiogram videos primarily containing the left and right coronary arteries (LCA and RCA, respectively). Algorithm 3 localized relevant objects within images of the LCA and RCA by outputting bounding box coordinates for identified objects (Supplementary Video). Coronary artery stenosis location was assigned according to greatest overlap between two Algorithm 3-predicted bounding boxes of the coronary artery sub-segment and stenosis (Fig. 1b). Algorithm 4 accepted images cropped around stenosed artery segments (by Algorithm 3 bounding boxes) and predicted the maximal percentage stenosis within the image as a continuous value between 0 and 100 for each image. Predictions were averaged across a video to provide the video-level prediction; and the mean of video-level predictions from all videos that visualized an artery segment within a study provided the final artery-level prediction.

### Algorithm 1: classification of angiographic projection angle

Algorithm 1 accepted individual images (video frames) as its input and identified the angiographic projection angle used. The projection angle refers to the fluoroscopic angulation used to obtain the image, commonly described on two axes defined by LAO-RAO and cranial-caudal (LAO cranial, RAO caudal, etc). For Algorithm 1 training data, all images from the extracted Full Dataset were categorized into 12 categories of left-right and cranio-caudal projection angles based on the primary and secondary angles extracted from each video’s metadata (−180 and 180 degrees for the primary angle and −50 and 50 degrees for secondary; Supplementary Table 1). We then split the extracted Full Dataset into training (990,082), development (128,590) and test datasets (299,625).

Algorithm 1 architecture was based on Xception, which is a convolutional neural network that has achieved state-of-the-art performance at image recognition tasks^33^. It was initialized with ‘ImageNet’ weights^34^, as commonly performed to initialize weights for faster algorithm convergence in image classification settings; all layers were trainable. Images were augmented by random zoom (range=0.2) and shear rotate (range=0.2). The development dataset was used to iteratively compare algorithm performance and fine tune hyperparameters using grid search (Supplementary Table 5). We experimented with different architectures such as VGG-16, ResNet50 and InceptionNet-V3 but found no incremental benefit over Xception. A grid search was used to fine-tune hyperparameters. The Test dataset was not used at all during training and was only used to report final performance. The most common prediction across extracted the frames of each video was assigned as the angiographic projection of that video; ties were addressed by selecting the projection with the highest average probability across all frames. We used Algorithm 1 weights as a ”core model” to initialize the weights for training the subsequent algorithms based on the Xception architecture.

### Algorithm 2: classification of primary anatomic structure

Algorithm 2 aimed to identify the primary anatomic structure present in an angiographic video (Supplementary Table 2), since it is common to capture angiogram videos containing non-cardiac anatomic structures such as the aorta or the femoral artery during the procedure. To generate Algorithm 2 training data, we randomly selected 14,366 images from the extracted Full Dataset, and a cardiologist categorized each image into one of 11 classes describing the primary anatomic structure (Supplementary Table 2). This dataset was split into Training/Development/ Test datasets, containing 9887 (70%), 1504 (10%), and 2975 (20%) images, respectively. We trained Algorithm 2 using the Xception architecture, initialized weights from trained Algorithm 1, and tuned hyper-parameters (Supplementary Table 5). Images were augmented by random zoom (range = 0.2) and shear rotate (range = 0.2). The predicted primary anatomic structure of a video was the mode prediction of all of its extracted Full Dataset frames. Only videos that primarily contained right or left coronary arteries flowed to Algorithm 3 for subsequent CathAI analyses (Supplementary Fig. 1). F1 scores and model performance varied by anatomic class, but in general classes with lesser frames had lower performance (Supplementary Fig. 3b), suggesting a possibility to improve performance if more labeled data were available.

### Algorithm 3: localization of angiogram objects

Algorithm 3 aimed to localize relevant objects within images of the left and right coronary arteries (the output of Algorithm 2). While Algorithm 3 was trained to localize multiple objects (Supplementary Table 3), the tasks most critical to the CathAI pipeline were to (i) identify coronary artery segments, (ii) identify stenoses (if present) and (iii) localize other relevant objects such as guidewires or sternotomy. To generate training data for Algorithm 3, 2338 contrast-containing images of LCA and RCA both with and without stenosis (as identified by Algorithm 2) were annotated by a cardiologist who placed bounding boxes around all relevant objects in the image (Supplementary Table 3). Only stenoses in the main epicardial vessels, not side branches such as diagonals or marginals, were labeled. In 100% of the 2338 frames, the LCA or RCA was the primary anatomic structure contained, and the artery was well visualized, well opacified, and not underfilled, according to the annotating cardiologist.

In our final CathAI pipeline we trained two versions of Algorithm 3: Algorithm 3a was trained on and accepted both LCA and RCA images as input. Since the RCA in the LAO projection contained the greatest number of annotated images in our dataset, we also trained a dedicated Algorithm 3b on this projection to demonstrate possible performance gains from focusing an algorithm on a specific artery/projection (RCA in LAO). To train Algorithms 3a/b, we split our labeled images for this task into two separate datasets: One containing left/right coronary arteries (2,338 images) and one containing RCA images in the LAO projection (450 images). Each dataset was subsequently split into 90% training (2,104 and 405 images respectively) and 10% test (234 and 45 images respectively) and Algorithms 3a/b were trained for 50 epochs. Once deployed in the CathAI pipeline, Algorithm 3b served to decrease input variability for Algorithm 3a, which produced performance improvements for both algorithms. Since we achieved performance gains by developing an algorithm on this specific artery/projection, future gains may be achieved with other dedicated algorithms.

Algorithms 3a/b employed the RetinaNet architecture and were trained using original hyperparameters^16^; a development dataset was not used. RetinaNet has achieved state-of-the-art performance for object localization such as the pedestrian detection for self-driving cars^35^. For our task, Algorithms 3a/b output bounding box coordinates for any objects present in each input image. Because some artery segments in certain angiographic projections are known a priori to be foreshortened or not visible, we applied a post-hoc heuristic to exclude certain Algorithm 3a/b-predicted artery segments from angiographic projections as predicted by Algorithm 1 (Supplementary Table 4). This thereby represents a fusion of intermediate predictions from two CathAI pipeline algorithms to achieve more clinically-relevant overall pipeline performance. To assess Algorithm 3a/b performance, the predicted coordinates were compared with the ground-truth coordinates using the ratio of the area of intersection over the area of union (called Intersection-over-union [IoU])^36^. An IoU≥0.5 between the predicted and annotated coordinates was considered a true positive. Next, we measured the mean average precision (mAP), which represents the ratio of true positives over true and false positives at different thresholds of IoU, for each class^37^ A mAP of 50% compares with state-of-the-art results for this type of task^16,35^.

### Algorithm 4: predicting the percentage of coronary artery stenosis

Algorithm 4 was developed to predict the severity of coronary artery stenosis as a percentage, given input images cropped around stenosed artery segments identified by Algorithm 3. Algorithms 3a/b were run on all Report dataset videos to localize artery segments and stenoses. All frames that contained a stenosis bounding box overlapping with a coronary artery segment bounding box with IoU ≥0.20 comprised potential input frames for Algorithm 4. A stenosis was localized to the artery segment that Algorithm 3 identified which had the greatest overlap by IoU. To derive train/test labels for Algorithm 4, we cross-matched stenoses found by Algorithm 3a/3b with the stenosis percentage found in the procedural report in corresponding artery segments (Supplementary Fig. 1). Matched procedural report values served as labels to train Algorithm 4 with input images cropped around stenosed artery segments according to Algorithm 3a/b bounding boxes. Non-matched stenoses were removed from our dataset. We also excluded all videos where an intra-coronary guidewire was present in more than 4 frames, as automatically determined by Algorithm 3a/b (6,076 videos, 41,780 stenosis-frames identified with guidewires), since these videos likely represent percutaneous coronary interventions which could alter the stenosis percentage within that video (Supplementary Fig. 1); videos were retained from studies prior to the insertion of an intracoronary guidewire. To train and validate Algorithm 4, we combined 6258 images of non-stenosed coronary artery segments with the remaining 98,756 images of stenoses.

Once a stenosis was identified, bounding box coordinates were expanded by 12 pixels in all dimensions, then cropped and resized to the nearest of three predetermined sizes: 256*256 pixels (aspect ratio no.1), 256*128 pixels (aspect ratio no.2) and 128*256 pixels (aspect ratio no.3). This was performed to maximize signal-to-noise (vessel-to-background) ratio, due to different vessel orientations and stenosis sizes. The “Report Dataset” used to train Algorithm 4 consisted of 105,014 images (6667 stenoses coming from 2,736 patients and 5,134 healthy vessel segments from 1,160 patients; Supplementary Fig. 1). Since non-stenosed vessel segments tended to be longer than focal stenosis which may bias training, we cropped all non-stenosed segments randomly to a height and width, mirroring the distribution of stenosis image sizes within that coronary segment. This yielded similar vessel sizes between the stenosed and non-stenosed images for each vessel segment. Images were randomly split into 70% training, 10% development and 20% in testing datasets.

Algorithm 4 was based on a modified Xception architecture where the last layer (Softmax layer, used for classification) was replaced with an ‘average pool’ then dense layer with a linear activation function to enable prediction of stenosis severity as a continuous percentage value. Image metadata consisting of the coronary artery segment label and cropped aspect ratios were also added as inputs into the final layer of Algorithm 4, which improved performance. The algorithm output a percentage stenosis value between 0 and 100 for every input image representing the maximal stenoses in that coronary artery segment. The percentage value was then averaged across all frames of the stenosed artery segment in a video, then averaged across videos of the same artery segment to obtain a final stenosis percentage (artery-level percentage).

Model weights were initialized using those from the trained Algorithm 1. Images were augmented by random flip (both horizontal and vertical), random contrast, gamma and brightness variations, random application of adaptive histogram equalization (To improve contrast in images). The algorithms were trained to minimize the squared loss between the predicted (AI-stenosis) and the report-stenosis using the RADAM optimizer^38^ with an initial learning rate of 0.001, momentum of 0.9 and batch size of 12, trained for 50 epochs. Training was halted when loss stopped improving for 8 consecutive epochs in the test dataset.

For Algorithm 4, we modified the training scheme such that each epoch was trained on images of one aspect ratio, with the next epoch training on another aspect ratio (copying all weights from the previous iteration), as performed previously for multi-size inputs^39^. This was iterated until convergence. We measured the algorithm performance on the complete test dataset, consisting of the three aspect ratios. We observed that the convergence of the multi-size input training was like other algorithms that used a fixed size for training. We also examined various pre-processing approaches and sequences without improvement in algorithm performance (Supplementary Table 7s).

### Neural network explainability methods

We applied two neural network explainability approaches to the fully-trained CathAI algorithms in order to better understand how algorithms made their predictions, respective to their relative tasks. The GradCAM^17^ technique highlights image regions most critical to prediction. Red highlighted areas denote higher importance to algorithm prediction, whereas more blue highlighted regions denote lower importance. We also derived saliency maps using the Layer Ordered Visualization of Information (LOVI) method^18^, which highlights individual pixels in the image that contribute most to algorithm predictions. Brighter pixels represent greater contribution to the algorithm’s prediction.

### External validation

For external validation, we randomly sampled 1000 coronary angiogram videos performed at the University of Ottawa Heart Institute (UOHI) between July 1st 2020 and October 31st 2020, acquired with Philips Azurion systems (Koninklijke Philips N.V., Amsterdam, Netherlands), at 15 fps, using Iopromide dye. Algorithms 1, 2 and 3 were applied to each video to identify and localize stenoses, and Algorithm 4 predicted AI-stenosis. We then sampled up to 40 examples of angiogram videos per coronary artery segment to form our external validation dataset, identifying a total of 464 coronary angiograms with distinct stenoses. Two board certified interventional cardiologists at the UOHI, each with over 2500 coronary angiograms of experience as primary operators, adjudicated these 464 videos in a blinded fashion by grading stenosis severity as a percentage between 0 and 100%, describing the underlying anatomic structure and localizing the stenosis to a coronary artery sub-segment. Algorithm performance in this dataset was reported as the AUC of the AI-stenosis compared to each adjudicator, and to the average of both adjudicators. Since stenoses in this external validation dataset were only visualized in one video, there was no calculation of artery-level AI-stenosis performance. The same binary threshold (0.54) was used for obstructive AI-stenosis as in the primary analysis. We also described the concordance between the localization of the stenosis as determined by Algorithm 3 and by the two adjudicators as well as the average difference between each adjudicator stenosis percentage.

To train CathAI Algorithm 4 for the different population distribution of the QCA dataset, we split the QCA dataset into training (75%), development (12.5%) and testing (12.5%) and fine-tuned the last two fully-connected layers of Algorithm 4, to allow the algorithm to learn to predict the QCA stenosis values from the input stenosis images rather than visually assessed stenosis. We performed a grid-search of initial learning rates from 1e^−4^ to 1e^−8^ and selected the rate which produced the lowest loss value on the development set in 100 epochs. We then trained the model starting with a learning rate of 1e-6 and dropping by a factor of 0.1 every 100 epochs, for 300 epochs.

### Quantitative coronary angiography dataset

The CathAI system provides an algorithmic foundation to be re-trained for future angiogram-relevant tasks. To demonstrate this, we obtained an external dataset of coronary angiograms of an a priori different patient population adjudicated at the Montreal Heart Institute (MHI) Core laboratory using the CMS QCA system (MEDIS, Leiden, Netherlands). This dataset was comprised of angiograms analyzed by the MHI Angiographic Core lab, obtained during randomized controlled trials (RCT) in ≥18 year old patients that had a coronary angiography intervention as part of the study, and received novel lipid lowering drugs or placebo^40^ The trials from which this data were derived used inclusion criteria that excluded patients with obstructive coronary artery disease (CAD) at the start of the study, which resulted in a majority of mild-to-moderate severity coronary stenosis in this dataset. QCA analysis was performed by two trained technicians and was supervised by an expert physician. For this dataset, severe stenosis was defined as ≥50% QCA stenosis severity^6,13^.

Coronary angiogram images were acquired using the Philips (Koninklijke Philips N.V., Amsterdam, Netherlands), General Electric Medical Systems (General Electric, Chicago, Illinois, United States) and Toshiba (Toshiba Corporation, Minato City, Tokyo, Japan) at 15 frames per second, by injection Iopromide dye into coronary arteries. For each QCA stenosis analysis at the MHI core lab, an end-diastolic frame was selected with angulations that best showed the stenosis at its most severe degree with minimal foreshortening and branch overlap. QCA software automatically calculated the percent diameter stenosis for coronary artery segments with reference diameter ≥1.5 mm.

This RCT QCA dataset contained a different patient population a priori from the real-world clinical UCSF dataset, since all stenoses ≥ 50% were not present during the baseline angiogram (but could be present at a follow-up angiogram), leaving primarily mild-CAD with a mean QCA stenosis severity of 31.7% ± 11.6%. This provided an optimal opportunity to examine how CathAI could provide a foundation for retraining using QCA stenosis labels to function as an automated tool for core lab angiogram analysis. We split the QCA dataset into training (75%), development (12.5%) and testing (12.5%) datasets and fine-tuned the last two fully connected layers of CathAI Algorithm 4 to predict QCA values rather than visually assessed stenosis estimates.

### Algorithm evaluation and statistical analysis

As appropriate for each task, each algorithm’s performance for categorical values was evaluated using positive predictive value (PPV), negative predictive value, sensitivity, specificity, area under the receiver operating characteristic curve (AUC), F1 score and Bland-Altmann plots. For continuous values, we present the intra-class (ICC [2,2])^41^ and Pearson correlation and the mean absolute error between CathAI’s stenosis prediction and the report stenosis or QCA stenosis. All three centers report continuous percentage stenoses as part of their routine clinical care.

Neural networks were trained using Keras v.2.24 and TensorFlow v.1.12. Final algorithms performance was reported in the Test Dataset. All analyses were performed using Python 2.7.

Algorithms 1 and 2 were evaluated on the frame/image level using precision (i.e. positive predictive value), recall (sensitivity) and plot the performance using confusion matrices. We also derived the F1 score for each class, which is the harmonic mean between the precision and recall.

To evaluate Algorithm 3a and 3b, we calculated the area of intersection over the area of union (IoU) between predicted bounding-box coordinates and the expert-annotated bounding-box coordinates of objects in each class in the test dataset. The IoU is the ratio between the area of overlap over the area of union between the predicted and annotated sets of coordinates^36^. An IoU≥0.5 signifies at least 50% area of overlap between the predicted and true bounding-boxes, which we considered a true positive. We then report the performance of Algorithm 3a/b as the mean average precision (mAP) metric, which represents the ratio of true positives over true and false positives at different thresholds of IoU, for every class^37^. A mAP value of 50% compares with state-of-the-art results for this type of task^16,35^. We also present the mean average precision for algorithm 3a and algorithm 3b by calculating the proportion of correct class prediction with an IoU≥0.1 with our ground-truth labeling across all our classes in our test dataset, as well as the positive predictive value of stenosis localization using the report or QCA dataset as ground truth.

To evaluate Algorithm 4, the primary metric of interest was the average absolute error between the reported value (REPORT-stenosis) and the predicted value (AI-stenosis) at the artery level. This mirrors guideline-based standard clinical practice for stenosis estimation, by measuring stenosis in multiple orthogonal projections and reporting the maximal degree of stenosis narrowing^2,3^. Image-level AI-stenosis was averaged across a video to obtain video-level AI-stenosis and compared against REPORT-stenosis using the mean squared error. Pearson and Intra-class correlation (ICC) and Bland-Altman^42^ plots were then calculated between the REPORT-stenosis and AI-stenosis at the video-level and artery-level. The reliability was classified as poor ( < 0.5), moderate (0.50–0.75), good (0.76-0.90), or excellent (0.91-1.0).(52) Finally, we present the mean squared error between REPORT-stenosis and AI-stenosis at the video-level.

To predict binary “obstructive” coronary artery stenosis, defined as ≥70% stenosis^2,3^, a threshold of 0.54 was used which optimized the F1 score. Based on this, we also report the area under the receiver operating characteristic curve (AUC), sensitivity, specificity and diagnostic odds-ratio^43^, at the frame level, video level and artery level, based on this threshold.

Confidence intervals for performance metrics were derived by bootstrapping 80% of the test data over 1000 iterations to obtain 5th and 95th percentile values. We present the performance of Algorithm 4 stratified by sex, by left and right coronary arteries, by artery segment and by age group. We also present two-sided *P* values for interaction (between the CathAI stenosis, the covariates and reported stenosis calculated by the Wald test). We categorized AI-stenosis and REPORT-stenosis in concordant and discordant lesion groups based on the visual ≥70% cutoff. For discordant lesions we present their prevalence, stratified by coronary artery segment. For lesion/vessel level data, a mixed effects logistic regression model as used to account for within-subject correlation and for repeated angiograms.

### Reporting summary

Further information on research design is available in the Nature Research Reporting Summary linked to this article.

### Supplementary information

Supplementary Material

Reporting Summary

## Data Availability

The data used in this study are derived from clinical care and thus are not made publicly available due to data privacy concerns. Reasonable requests for collaboration using the data can be made from the authors, as feasible and permitted by the Regents of the University of California.

## Code availability

The code that supports this work is copyright of the Regents of the University of California and can be made available through license. The data used to train and validate the algorithm cannot be made publicly available as it contains personal health information.
