# Supplementary material for: CathAI: fully automated coronary angiography interpretation and stenosis estimation
Source: NPJ Digit Med. 2023 Aug 11;6:142. doi: 10.1038/s41746-023-00880-1 (PMC10421915; doi:10.1038/s41746-023-00880-1)
Supplement: Supplementary file 1 — Supplementary Material [file 41746_2023_880_MOESM1_ESM.pdf]

SUPPLEMENTARY MATERIAL

Supplementary Discussion:

The CathAI pipeline requires ~2 seconds to analyze each video on consumer-grade GPUs (NVIDIA GTX 1080 Ti). Algorithm 1 performance was worse in the less commonly used antero-posterior and right anterior oblique (RAO)-lateral projections, and on the heterogeneous "other" class, defined as images that were not members of other listed classes. For Algorithm 2, more training data was generated for left and right coronary arteries in order to achieve improved performance, since these angiogram videos were critical to subsequent algorithms in the pipeline.

To better understand the Algorithm 3-predicted stenoses that were unmatched to REPORT-stenoses and excluded from our dataset, an interventional cardiologist manually reviewed 250 stenosis videos where Algorithm 3 identified stenoses in >7 frames. In the majority of these, Algorithm 3 identified an angiographic stenosis (86.4%, n=216) that could be visually appreciated. Most were described qualitatively in the clinical report such as "moderately stenosed", which could not be accurately assigned a stenosis percentage through our report text parsing approach (41.6%, n=104), while 12.0% (n=30) were not described at all in the clinical report. In 10% (n=25), the stenoses were in a marginal or diagonal branch which Algorithm 3 was not trained to identify. The report extraction of stenosis percentage failed in 8.8% (n=22), due to having a concomitant description of stenoses percentage in unsupported segments, such as diagonals. Algorithm 3 also exhibited several errors: 9.2% (n=23) stenoses where the stenosis was assigned to the wrong artery segment, 4.8% (n=12) where the stenosis was assigned to the wrong coronary artery (mainly LAD stenoses assigned to left main or left circumflex stenoses assigned to the LAD) and 13.6% (n=34) where Algorithm 3 falsely identified a stenosis due, for example, to vessel tortuosity being misinterpreted as a stenosis.

We examined the characteristics of those patients who were determined to have obstructive AI-stenosis (</≥70%) that were either concordant (1,336) or discordant (398) with the REPORT-stenosis (Extended Figure 9). We specifically refrain from using the terms “false positive/false negative” in this setting, since as we discussed, the REPORT-stenosis is subject to error and non-trivial variability. AI-stenosis was more likely to be discordant with REPORT-stenosis in older patients (62.7±13.2 vs 65.1±12.3, <0.001), in the LCA, the proximal RCA, distal RCA, the right posterolateral and the distal LAD.

To examine the performance of our SSIM-based approach to identify the peak-contrast frame, we examined the ratio of the peak-contrast frame number over the total frames in a video. Since contrast dye injection usually begins within the first 0.5 seconds of the typical 4-6 second angiogram video, we would expect most videos to exhibit their peak-contrast frame within the first 20-50% (ratio 0.2-0.5) of frames. Across the Full Dataset, the SSIM-selected peak-contrast frame ratio was 0.33±0.18 (Extended Figure 10), which is consistent with expectation.

The current state-of-the-art for assisted coronary stenosis assessment primarily encompasses QCA[1], which still relies upon significant human input and exhibits significant variability. A study assessing 10 different QCA systems against a phantom stenosis gold-standard found absolute percentage differences of -26% to +29% in coronary stenosis assessments between systems[2]. For different individuals using the same QCA system an 11.2% absolute percentage difference was reported. Notably, on our post-hoc review of examples of angiograms where AI-stenosis and REPORT-stenosis were discordant, the REPORT-stenosis value was not always objectively more correct. Because the mean absolute difference between AI-stenosis and REPORT-stenosis of ~18% was so similar to human inter-observer variability[3,4](which is also likely present in the clinically-generated Report dataset), CathAI

performance may be substantially improved if re-trained using less variable training labels—such as with core lab-generated QCA estimates. Therefore, QCA or other stenosis assessment assistance methods could play an important role to provide large numbers of high-quality training labels with which to further improve CathAI. Similarly, training dedicated algorithms for certain higher-volume angiographic projections, like our Algorithm 3B, could decrease input variability into all models and improve overall performance.

The model explainability methods we performed highlight how individual algorithms in CathAI function to accomplish their tasks. GradCAM and LOVI suggest that the algorithms often focused on similar regions of the image as a human expert does to classify anatomy and predict stenosis severity. For stenosis assessment (Algorithm 4), LOVI showed that CathAI involved pixels not only at the narrowest part of the stenotic artery but also outside of the area of stenosis, like how human cardiologists use both normal and abnormal segments of the coronary artery to assess relative severity.

CathAI's could also be trained with additional labels to define overall CHD burden, like an automated SYNTAX score[5], to guide revascularization decisions. The CathAI pipeline provides a foundation upon which a wide range of clinically relevant applications related to automated angiographic analysis can be trained with different task-specific labels. For example, CathAI could be trained to predict FFR or IFR values directly from angiographic videos containing stenosis or to highlight poorly-visible objects like prior stents, collateral arteries or bypass graft sites.

Additional limitations include that the text-parsing method we used to extract the REPORT-stenosis from the clinical procedure report may have introduced errors in either the location of the stenosis or the degree of severity. Resultant variability in stenosis labels used for

training, either from clinical variability or parsing, would be expected to adversely impact algorithm performance by biasing results to the null and decreasing the observed effect of association. Perhaps in part due to this variability in the stenosis training labels, our algorithm replicated the clinical bias and tended to underestimate severe stenoses and overestimate minor stenoses. This may also have been impacted, in part, by having fewer training examples of severe stenosis.

Supplementary Figures

Supplementary Figure 1.

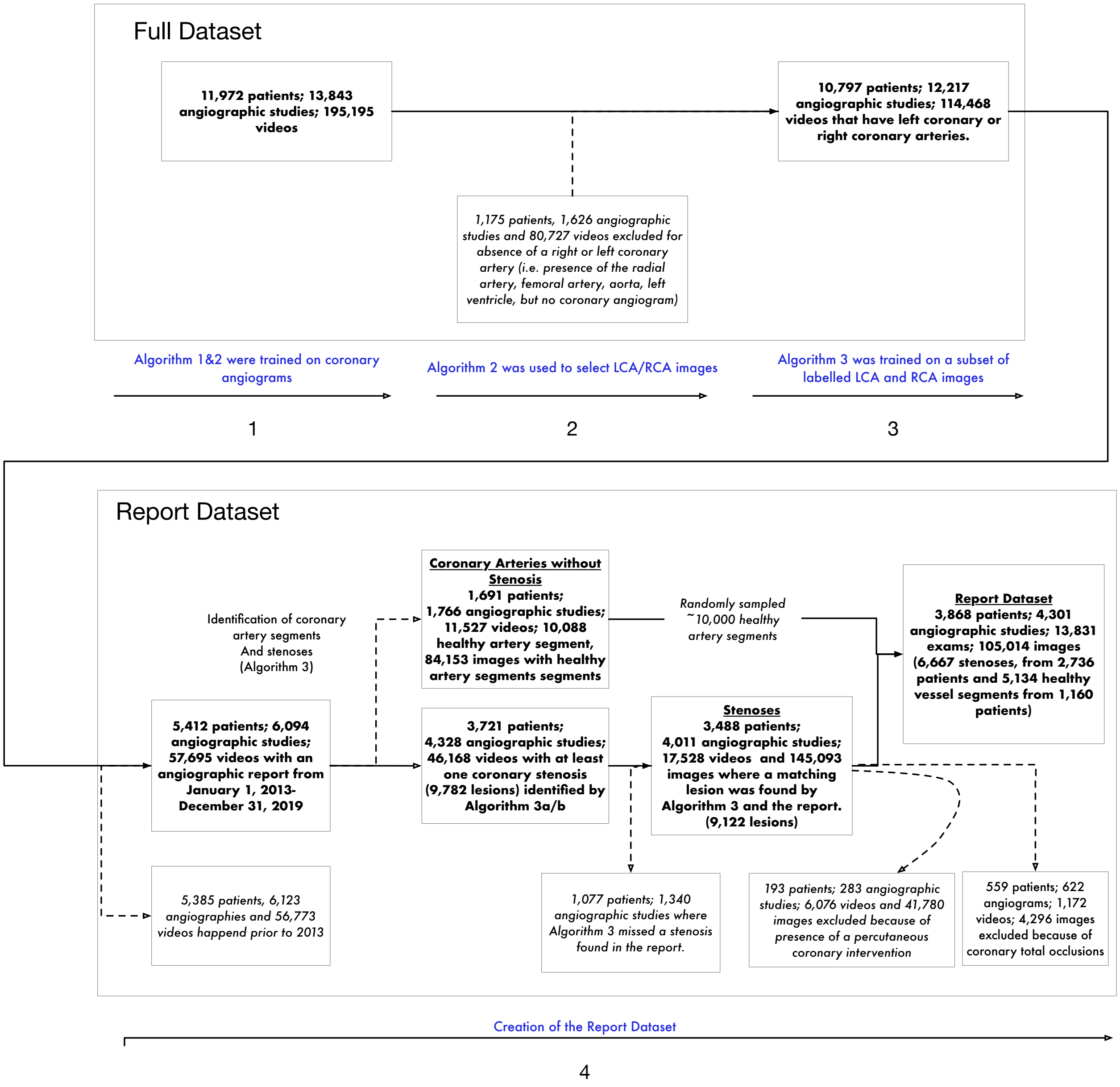

z

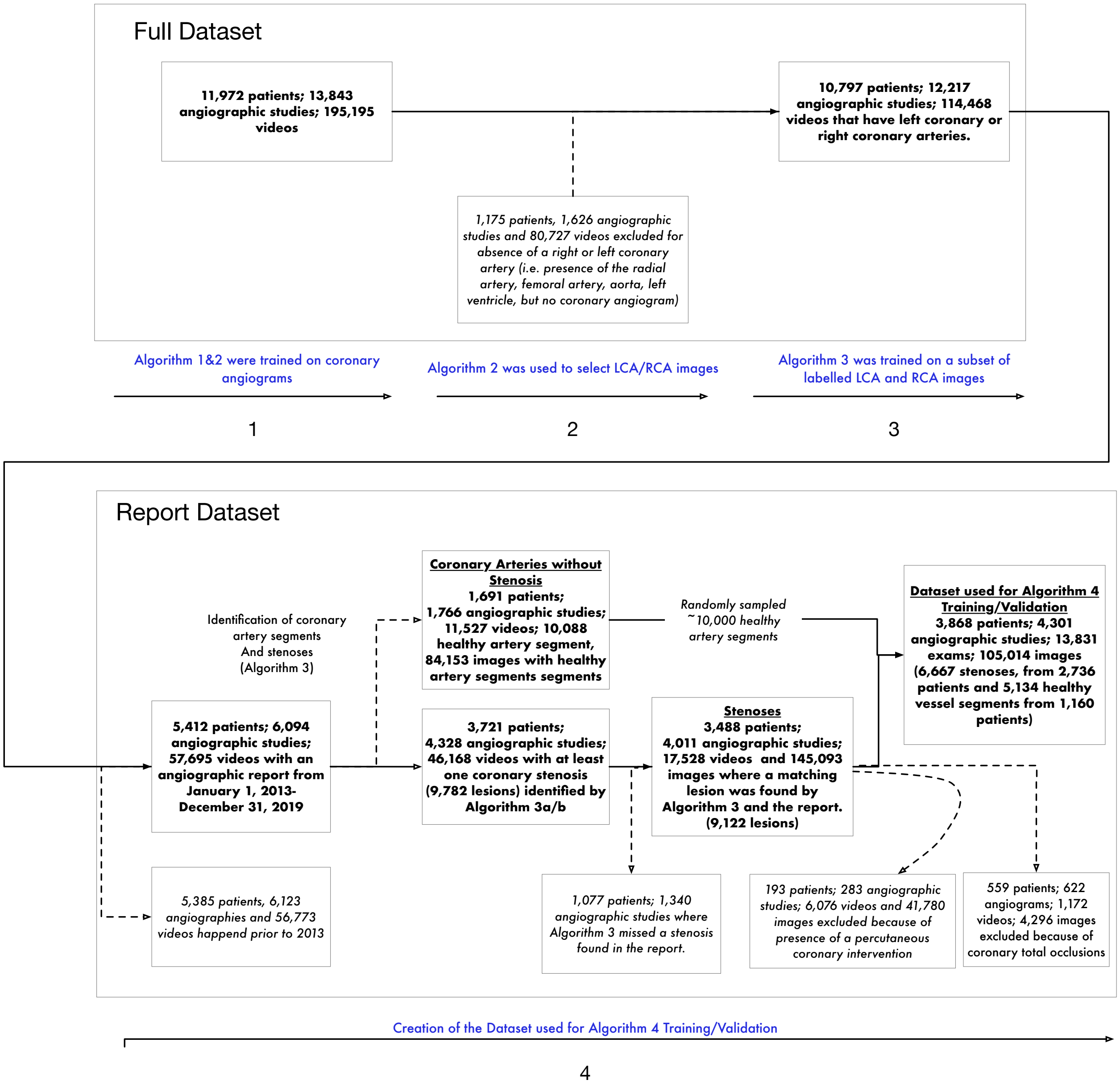

Supplementary Figure 1. Datasets and patients used to develop CathAI algorithms. Detailed description of the Full Dataset and the Report Dataset. Step 1: We trained Algorithm 1 on the Full Dataset and Algorithm 2 on a subset of the Full Dataset to identify angiographic projections and anatomical structures, respectively. Step 2: We applied Algorithm 2 to the Full dataset to identify videos with left or right coronary arteries. Step 3: We selected left or right coronary artery videos and randomly selected a subsample to manually annotate for Algorithm 3 training. Step 4. To generate the Report Dataset, only angiographic studies associated with a digital procedural report

were retained (those performed after 2013). We then cross matched the studies with a procedural report with stenosis identified by Algorithm 3. **Full arrows:** Creation of the study datasets; **Dashed arrows:** Excluded data. Abbreviations: LCA: Left Coronary Artery, RCA: Right Coronary Artery.

Supplementary Figure 2.

a.

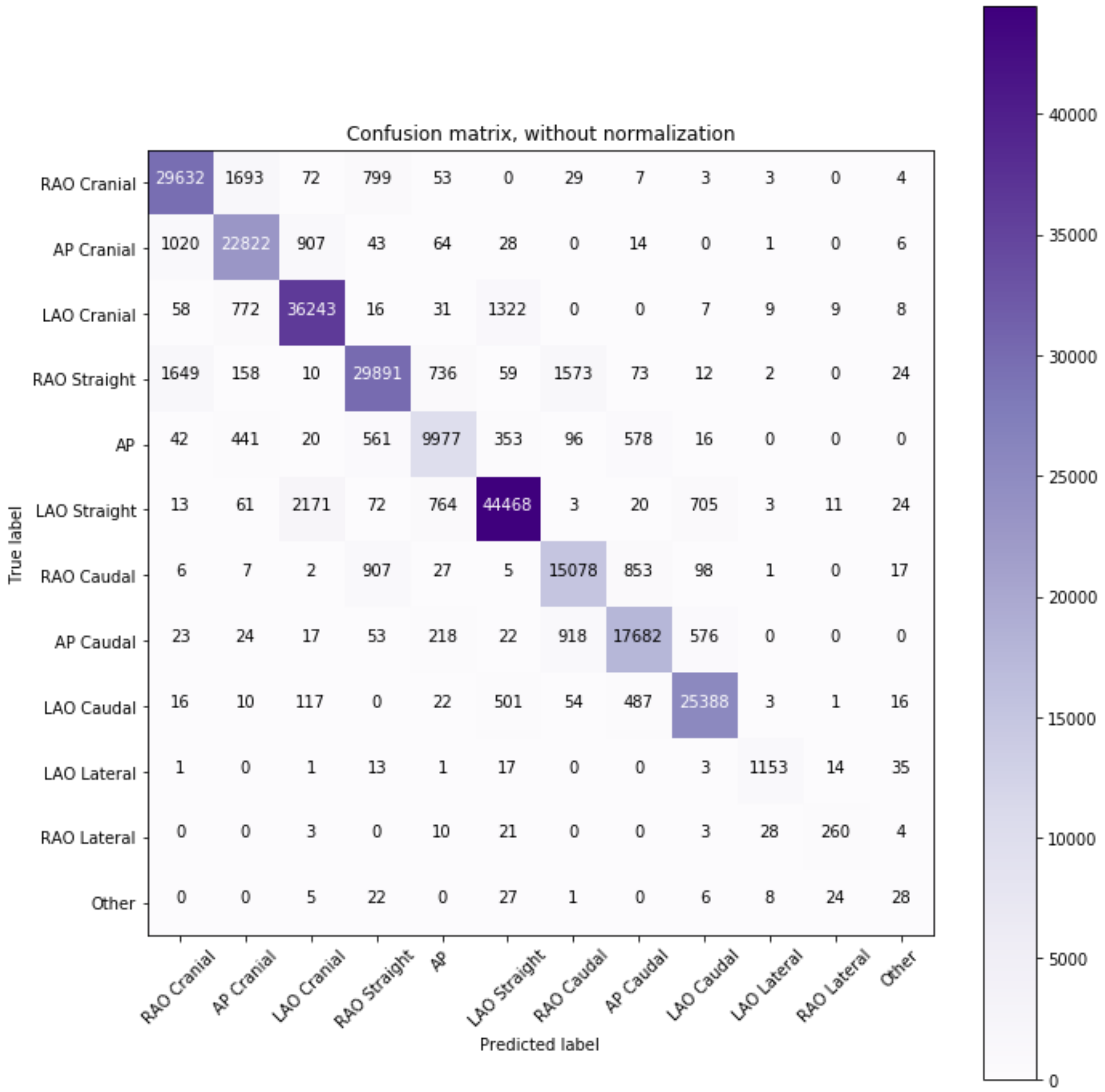

b.

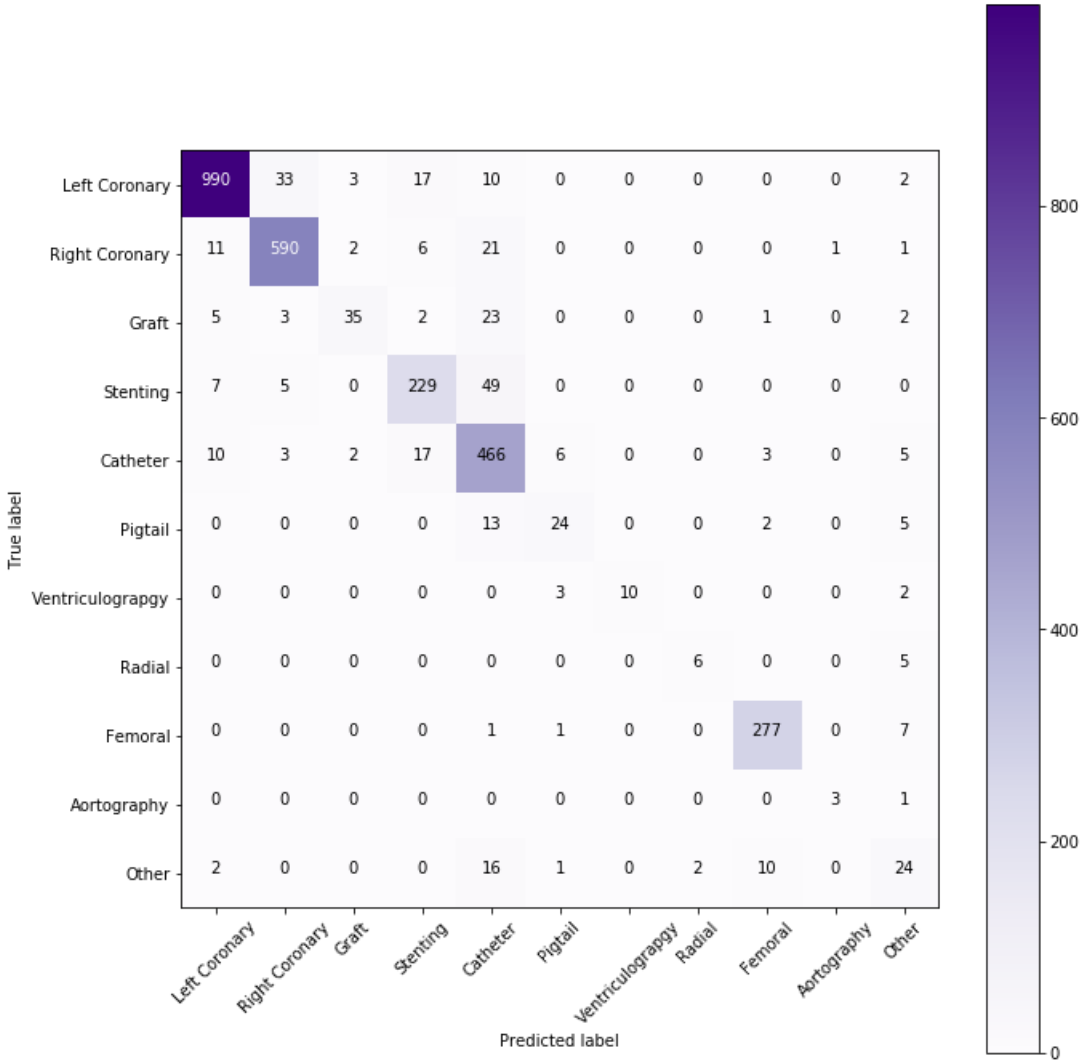

Supplementary Figure 2. Confusion matrices for classification of angiographic projection angle and anatomic structure at the frame-level. a. Algorithm 1 - Confusion Matrix for classification of angiographic projections at the frame-level in the test dataset. **b.** Algorithm 2 - Confusion Matrix of the classification of the different anatomic structures, at the frame level in the test dataset. Abbreviations: RAO: Right Anterior Oblique; AP: Antero-posterior; LAO: Left Anterior Oblique. Square color intensity correlates with the value within each square.

Supplementary Figure 3. Bland-Altman analyses between AI-stenosis and REPORT-stenosis

a.

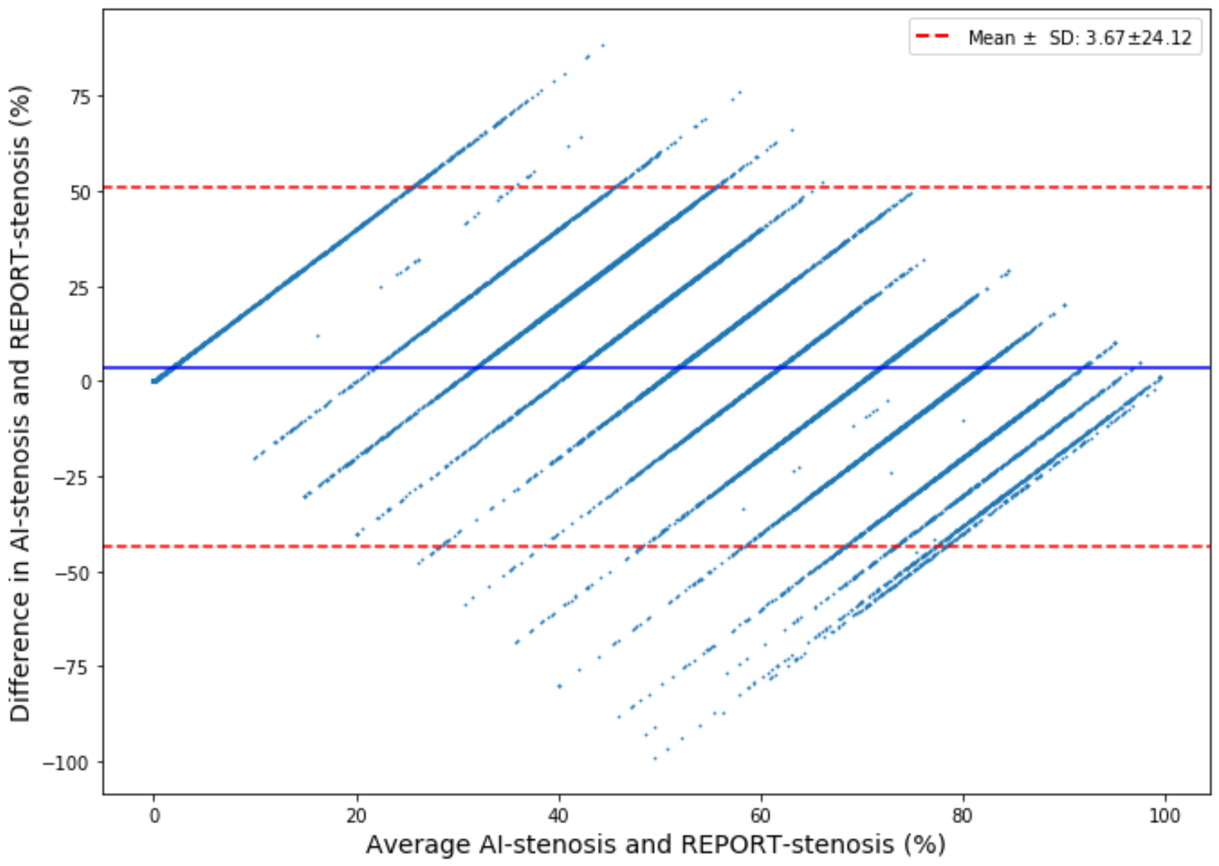

b.

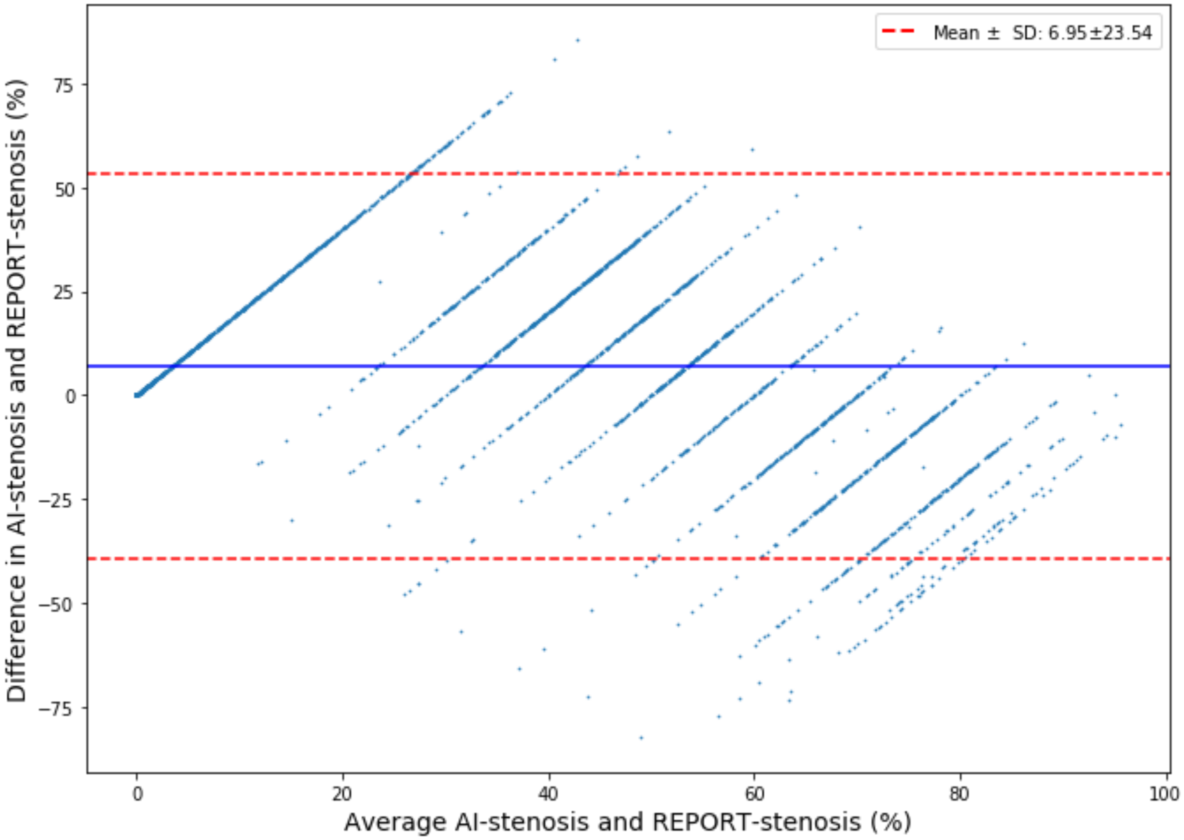

Mean ± SD: 6.95±23.54
75
50
25
0
−25
−50
−75
Difference in AI-stenosis and REPORT-stenosis (%)
0
20
40
60
80
100
Average AI-stenosis and REPORT-stenosis (%)

c.

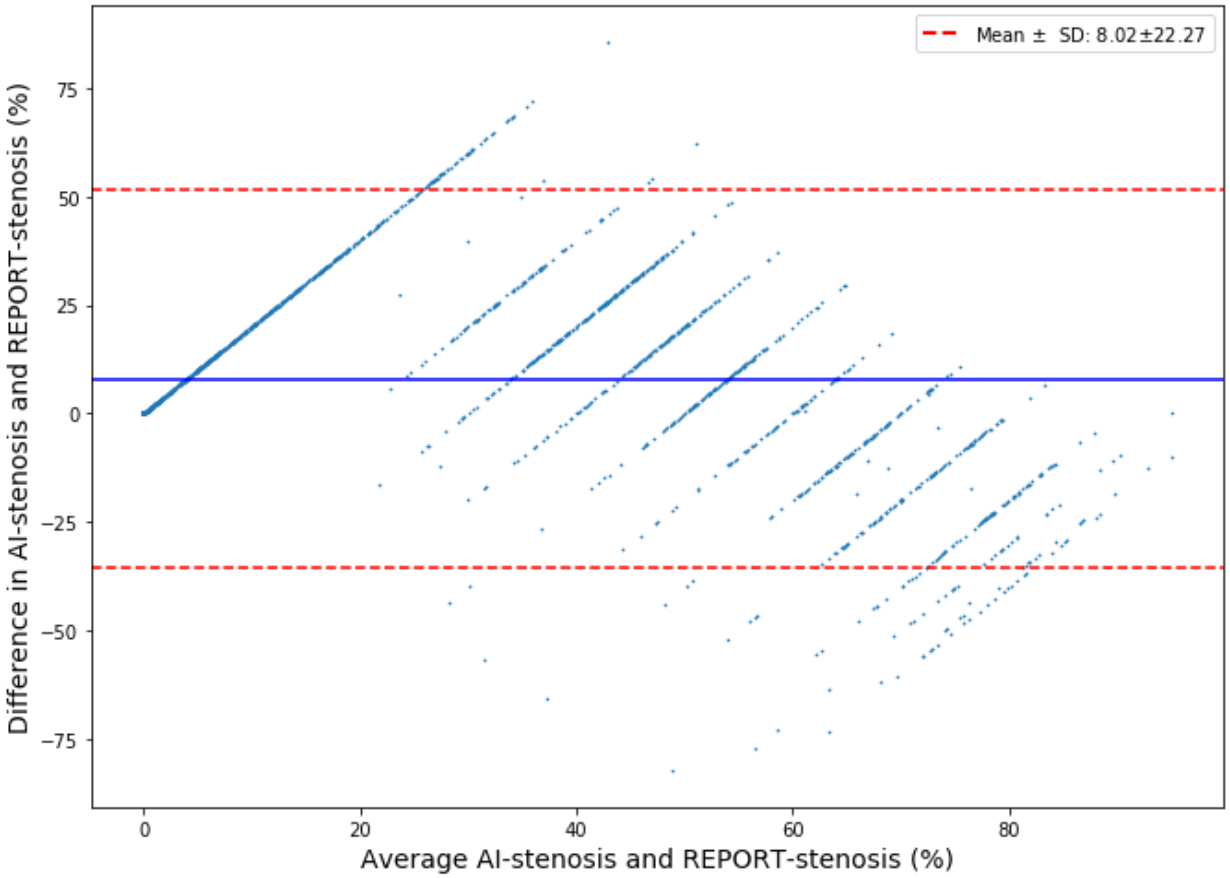
Mean ± SD: 8.02±22.27
75
50
25
0
−25
−50
−75
Difference in AI-stenosis and REPORT-stenosis (%)
0
20
40
60
80
Average AI-stenosis and REPORT-stenosis (%)

Supplementary Figure 3. Bland-Altman analyses between AI-stenosis and REPORT-stenosis. a. Difference between AI-stenosis and REPORT-stenosis at the artery-level for Algorithm 4a. **b.** Difference between AI-stenosis and REPORT-stenosis at the video-level for Algorithm 4a. **c.** Difference between AI-stenosis and REPORT-stenosis at the frame-level for Algorithm 4a.

Supplementary Figure 4. Confusion matrix of CathAI prediction of angiographic projection angle at the image-level in the external validation dataset (Algorithm 1)

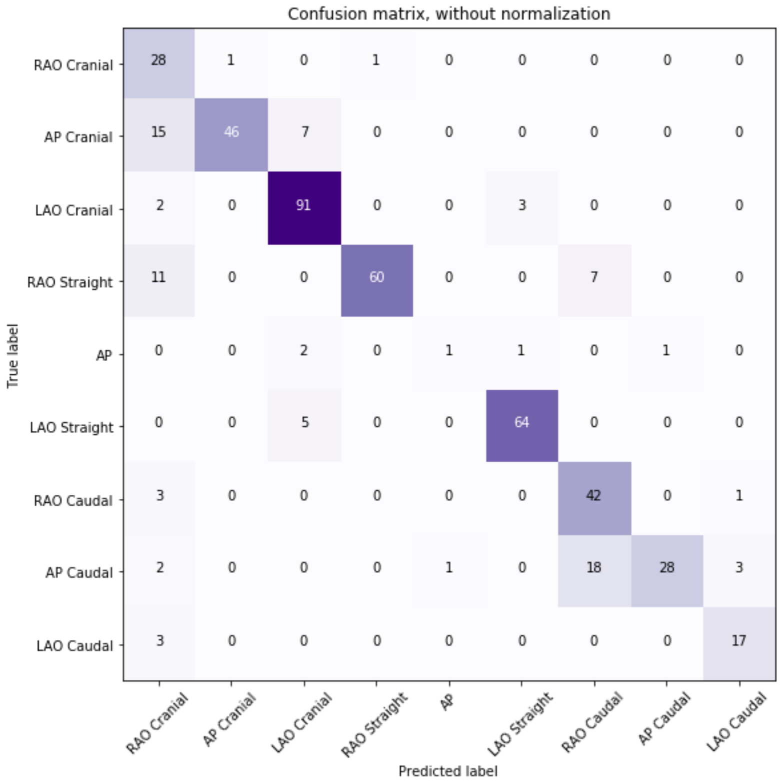

Supplementary Data Figure 4. Confusion matrix of CathAI prediction of angiographic projection angle at the image-level in the UOHI external validation dataset (Algorithm 1). Abbreviations: RAO: Right Anterior Oblique; AP: Antero-posterior; LAO: Left Anterior Oblique. Square color intensity correlates with the value within each square.

Supplementary Figure 5. Bland-Altman analyses between AI-stenosis and QCA-stenosis

a.

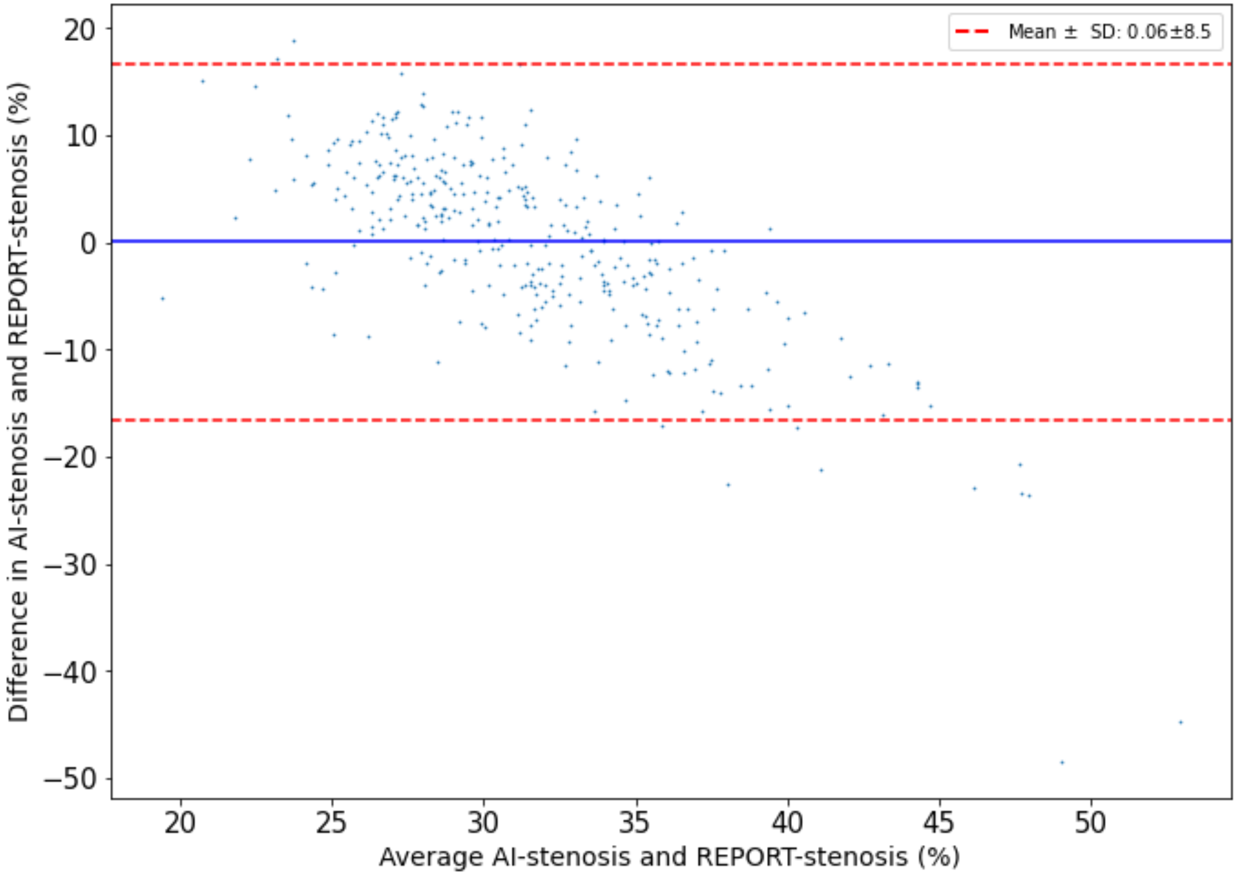

b.

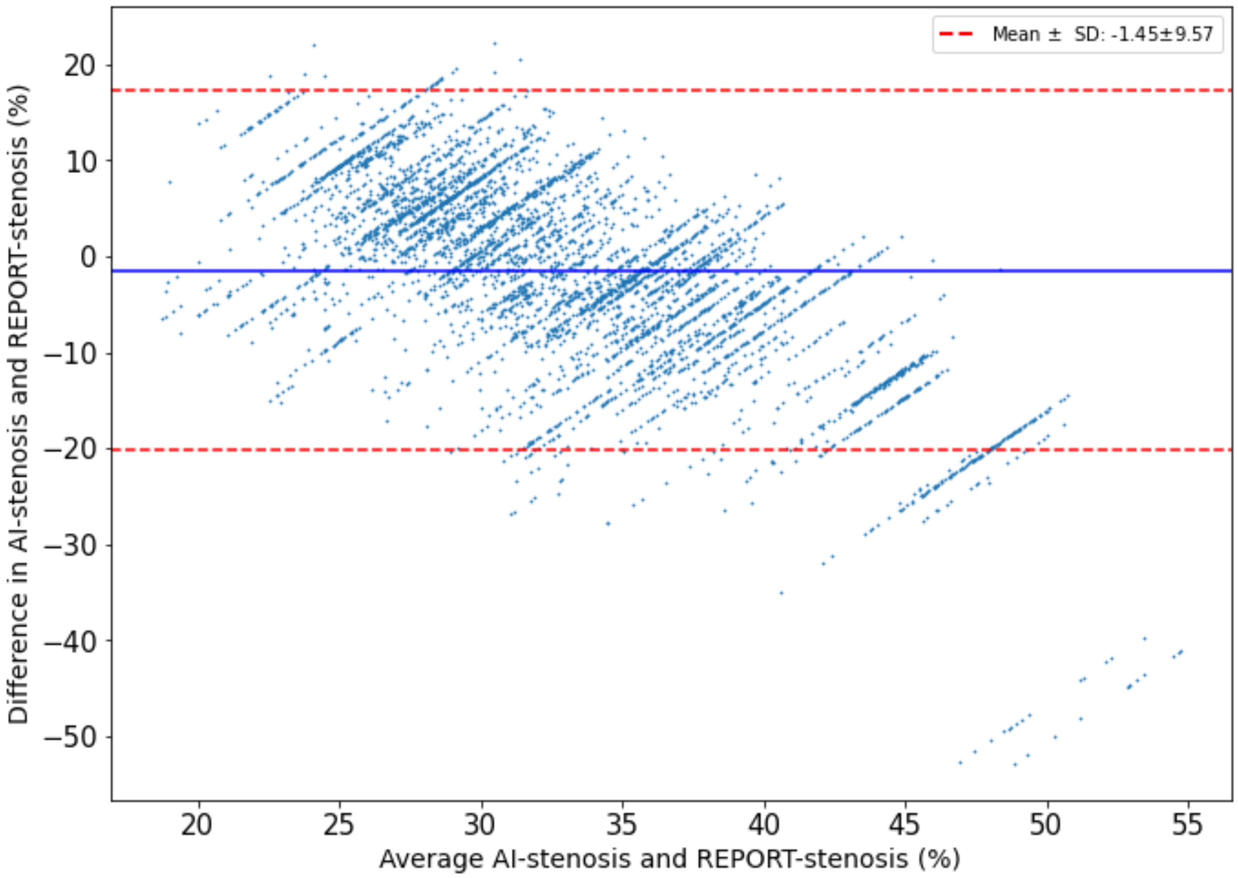

Supplementary Figure 5. Bland-Altman analyses between AI-stenosis and QCA-stenosis
a. Difference between AI-stenosis and QCA-stenosis at the artery-level on the fine-tuned algorithm for Algorithm 4a. **b.** Difference between AI-stenosis and QCA-stenosis at the frame-level on the fine-tuned algorithm for Algorithm 4a.

Supplementary Figure 6. LOVI saliency maps for classification of angiographic projection angle (Algorithm 1).

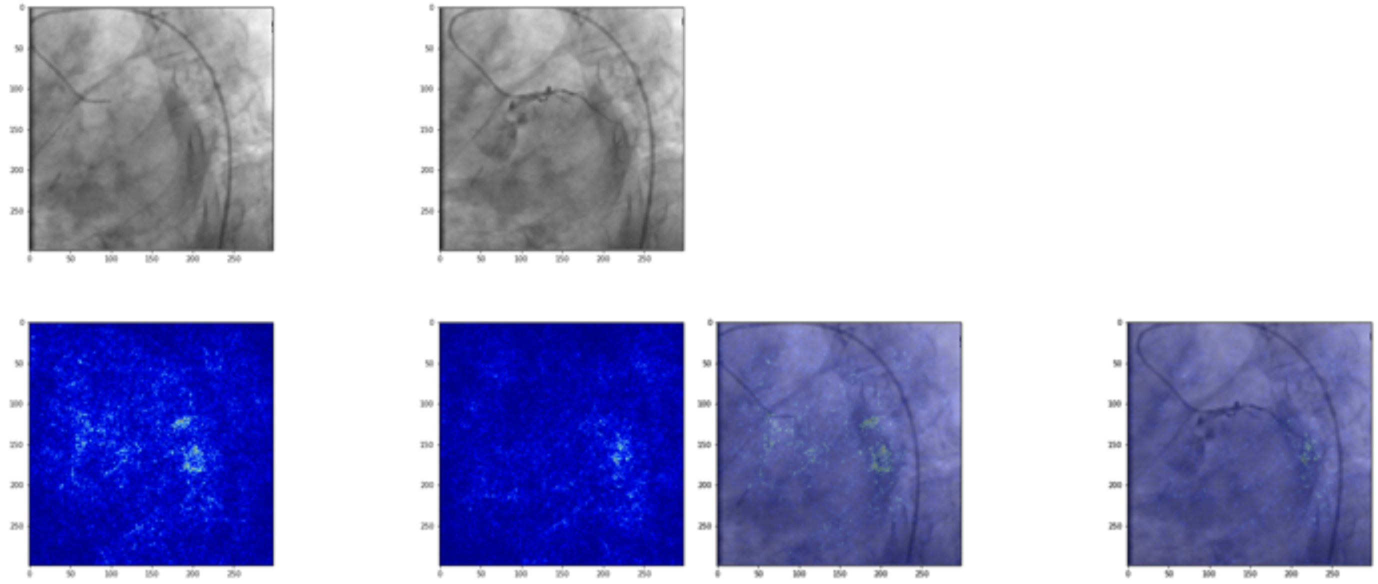

Supplementary Figure 6. LOVI saliency maps for classification of angiographic projection angle (Algorithm 1). Original angiogram images are shown (top). Corresponding saliency maps (middle) show brighter pixels representing greater contribution to Algorithm 1's prediction of angiographic projection angle. LOVI saliency maps overlaid on the original image (bottom). LOVI: Layer Ordered Visualization of Information

Supplementary Figure 7. Distribution of peak contrast frame selection

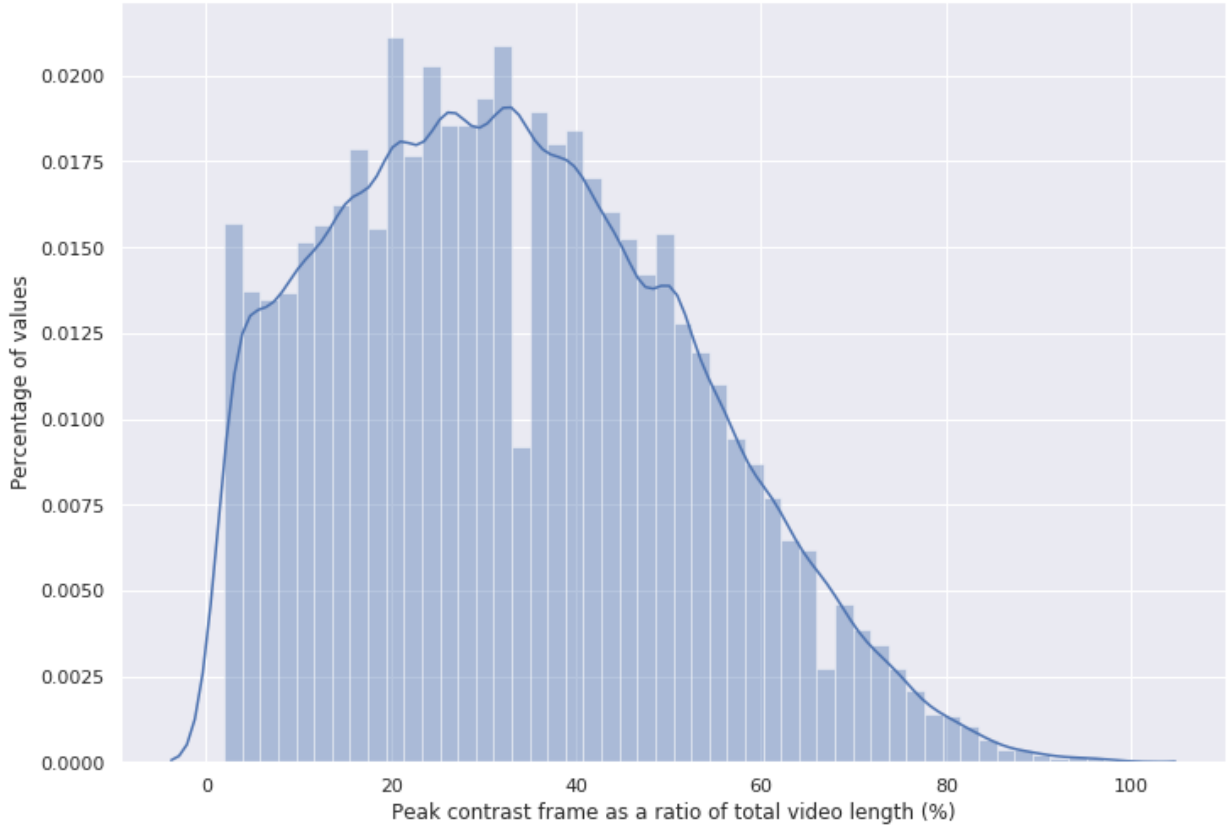

Supplementary Figure 7. Distribution of the frame selection approach using the structural similarity index measure (SSIM) calculated on all videos from the index frame ‘0’ as a ratio of the total video length. Formula used was : Peak Contrast Frame Ratio = (Video Duration - Peak Contrast Frame)/(Video Duration). This demonstrates that our heuristic of identifying the peak contrast frame using SSIM selects frames on average at 33.3±18.8% of the video length. This is consistent with expectation, since contrast dye injection usually begins within the first 0.5 seconds of the typical 4-6

second angiogram video, leading most LCA/RCA videos to exhibit their peak-contrast frame within the first 20-50% (ratio 0.2-0.5) of video frames. Non LCA/RCA videos are more likely to have their “peak contrast” frame selected from other (early and late) regions of the video.

Supplementary Figure 8. Data Annotation Examples

a.

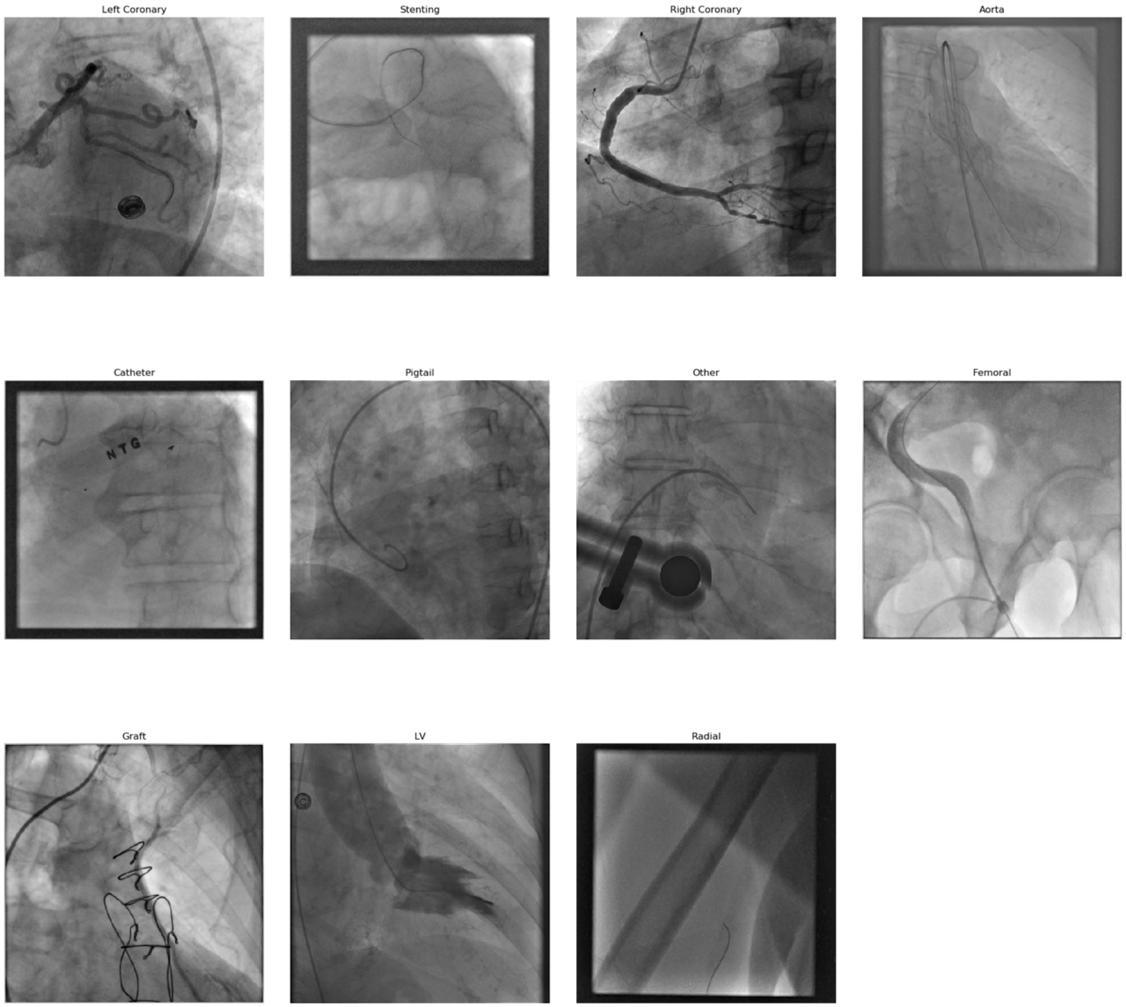

b.

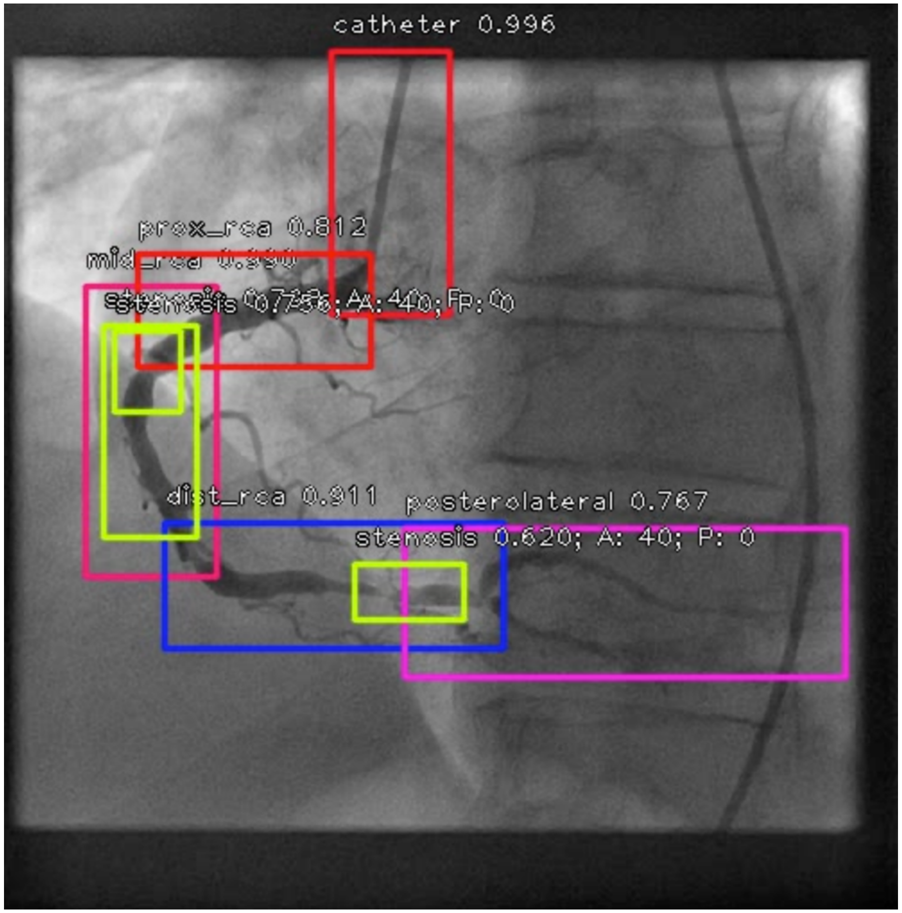

Supplementary Figure 8 Legend. a. For Algorithm 2, the peak contrast frame was labelled into one of the 11 categories by a board-certified cardiologist to allow the model to learn the underlying anatomical structure. **b.** For Algorithm 3, bounding boxes were drawn around catheters, artery segments (as defined in SYNTAX) and for stenoses. Stenoses bounding boxes start and ended in healthy segments of the vessel.

Supplementary Video

This video shows an example of CathAI functioning to localize objects in a real-world coronary angiogram. Raw Algorithm 3 output is shown first, followed by an annotated version highlighting several identified objects.

Supplementary Tables

Supplementary Table 1. Class Definitions of Angiographic Projection Angle Used for Algorithm 1

Class	Definition
RAO Cranial	-45° to -15° RAO; 15° to 45° Cranial
AP Cranial	-15° to 15° AP; 15° to 45° Cranial
LAO Cranial	15° to 45° LAO; 15° to 45° Cranial
RAO Straight	-45° to -15° RAO; -15° to 15° AP
AP	-15° to 15° AP; -15° to 15° AP
RAO Caudal	-45° to -15° RAO; -45° to -15° Caudal
AP Caudal	-15° to 15° AP; -45° to -15° Caudal
LAO Caudal	15° to 45° LAO; -45° to -15° Caudal
LAO Straight	15° to 45° LAO; -15° to 15° AP
LAO Lateral	70° to 110° LAO; -15° to 15° AP
RAO Lateral	-110° to -70° RAO; -15° to 15° AP
Other	Any angles not belonging to the previous definitions

Abbreviations: RAO: Right Anterior Oblique; AP: Antero-posterior; LAO: Left Anterior Oblique.

Supplementary Table 2. Classes of Primary Anatomic Structures Used for Algorithm 2

Class	Definition
**Left coronary artery**	Artery that arises from the aorta above the left cusp of the aortic valve
**Right coronary artery**	Artery that arises from the aorta above the right cusp of the aortic valve
**Bypass Graft**	Venous graft, internal mammary graft or radial graft
**Catheter**	Any guiding catheter or diagnostic catheter without any other underlying structure
**Pigtail Catheter**	Pigtail catheter without any other underlying structure
**Left ventricle**	Ventricle, as delimited during ventriculography
**Aorta**	Ascending aorta, the arch or descending aorta, as delimited during aortography
**Radial Artery**	Major artery in the forearm
**Femoral Artery**	Either the superficial, deep or common femoral artery
**Other**	Any images not belonging to the other classes (for example, kidneys, pacemaker, etc)

Supplementary Table 3. Definition of Objects Used for Algorithm 3

Class	Definition
**Coronary segment**	
Proximal RCA*	From ostium to one half the distance to the acute margin of the heart.
Middle RCA*	From end of first segment to acute margin of heart.
Distal RCA*	From the acute margin of the heart to the origin of the posterior descending artery.
Posterior descending artery*	Artery running the posterior interventricular groove.
Posterolateral artery*	Posterolateral branch originating from the distal coronary artery distal to the crux. If left posterolateral, it was chosen as the artery running to the posterolateral surface of the left ventricle.
Left main artery*	From the ostium of the LCA through bifurcation into left anterior descending and left circumflex branches.
Proximal LAD*	Vessel between left main and proximal to and including the first septal
Middle LAD*	LAD immediately distal to the origin of first septal branch and extending to the point where the LAD forms an angle (RAO projection). If angle is not identifiable, this segment ends at one half the distance form the first septal and the apex of the heart
Distal LAD*	Terminal portion of LAD, beginning at the end of previous segment and extending to or beyond the apex.
Proximal LCX*	Main stem of circumflex from its origin of left main to and including origin of first obtuse marginal branch.
Distal LCX*	The stem of the circumflex distal to the origin of the most distal obtuse marginal branch and running along the posterior left atrioventricular grooves. Caliber may be small or artery absent.
**Other classes**	

Valve	Presence of a mechanical valve, annuloplasty or valvular calcifications
Catheter	Presence of a catheter, such as a diagnostic catheter, pigtail or guiding catheter
Sternotomy	Presence of sternotomy wires
Stent	Stent landmarks on a guidewire or in a vessel
Pacemaker	Presence of a pacemaker or pacemaker lead
Guidewire	Presence of a guide wire
Stenosis	Any visible stenosis that is also described in the cath report.
Obstruction	100% obstruction of an artery, either by thrombus or chronically occluded. Defined by a blunt stump at the end to a vessel or by the ‘absence’ of contrast in between two healthy vessel segments with bridging collaterals.

Abbreviations: RCA: Right Coronary Artery; LAD: Left Anterior Descending Artery; LCX: Left Circumflex.

*These coronary vessel segments follow the SYNTAX score definition.(41)

Supplementary Table 4. Heuristic Exclusion of Coronary Artery Segments by Angiographic Projection

Projection	Excluded segments	
	Right Coronary Artery	Left Coronary Artery
**RAO Cranial**	None	Proximal LCx, Distal LCx
**AP Cranial**	None	Proximal LCx, Distal LCx
**LAO Cranial**	None	Proximal LCx, Distal LCx
**RAO Straight**	Proximal RCA	Proximal LAD
**AP**	None	Mid LAD, Distal LAD, Distal LCx
**RAO Caudal**	None	Mid LAD, Distal LAD
**AP Caudal**	None	Distal LAD
**LAO Caudal**	None	Mid LAD, Distal LAD
**LAO Straight**	None	Proximal LCx, Distal LCx
**LAO Lateral**	None	None
**RAO Lateral**	None	None
**Other**	None	None

Supplementary Table 5. Hyperparameters searched for Algorithms 1 and 2

Hyperparameters	Values
**Learning Rate**	10e-2; 10e-3; 10e-4
**Architecture**	ResNet50
	Xception
	InceptionNet_V3
	VGG16
**Early stopping criteria**	4, 8, 16
**Optimizer**	Adam
	RAdam

Supplementary Table 6. Hyperparameters searched for Algorithm 4

Hyperparameters	Values
**Learning Rate**	10e-2; 10e-3; 10e-4
**Architecture**	Xception with additional inputs: ‘image size’
	Xception with additional inputs: ‘image size’, ‘artery segment’
	Xception with additional inputs: ‘image size’, ‘artery segment’, ‘angiographic projection’
	Xception
	InceptionNet_V3
	ResNet50
	VGG16
**Early stopping criteria**	4, 8, 16
**Optimizer**	Adam
	RAdam
**Lookahead**	True
	False
**Image augmentation**	1. Rotation, horizontal flipping and vertical flipping<br>2. Rotation, horizontal flipping and vertical flipping, contrast and brightness adjustment<br>3. Rotation, horizontal flipping and vertical flipping, contrast and brightness adjustment, scaling, shearing and random cropping

**Datasets**	1. Stenoses bounding boxes<br>2. Stenoses bounding boxes expanding by 12 pixels<br>3. Stenoses bounding boxes resized to a fixed 256x256 size<br>4. Stenoses bounding boxes centered in a 256x256 image, with zero-padded borders<br>5. Segmented stenoses

Supplementary Table 7. Development set performance of alternative training schemes and hyperparameters examined for Algorithm 4

Alternate Training Scheme Name	Hyperparameters tuned	Best development set performance at the at the image level	Best development set performance at the at the artery level
**Selected algorithm**			
**Three different aspect ratios of images, with 12 px. Offset around the vessel, enriched with 10000 healthy vessel images, excluding certain vessel segments from certain views**	Three frames as input, with three aspect ratios<br>Learning rate {1e-3, 1e-4, 1e-5}<br>Mean squared error optimizer<br>Batch size 12<br>RAdam optimizer + Lookahead<br>Max Epochs 50<br>Image augmentation (rotation, mirroring, scaling)	0.76	0.86
**Fixed size 256x256 images of non-segmented vessels, enriched with 10000 healthy vessel images**	Three frames as input<br>Learning rate {1e-3, 1e-4, 1e-5}<br>Batch size 12<br>Adam optimizer + Lookahead<br>Root mean squared error optimizer<br>Max Epochs 50<br>Image augmentation (rotation, mirroring, scaling)	0.72	0.80

**Zero padded images of stenoses enriched with 10000 zero padded healthy vessel images**	Three frames as inputLearning rate {1e-3, 1e-4, 1e-5}<br>Mean squared error optimizer<br>Batch size 12<br>Adam optimizer + Lookahead<br>Max Epochs 50<br>Image augmentation (rotation, mirroring, scaling)	0.63	0.63
**Three different aspect ratios of images, with 12 px. Offset around the vessel, enriched with 10000 healthy vessel images**	Three frames as input, with three aspect ratios<br>Learning rate {1e-3, 1e-4, 1e-5}<br>Root Mean squared error optimizer<br>Batch size 12<br>Adam optimizer + Lookahead<br>Max Epochs 50<br>Image augmentation (rotation, mirroring, scaling)	0.75	0.85
**Three different aspect ratios of images, with 12 px. Offset around the vessel, enriched with 10000 healthy vessel images**	Single frame as input, with three aspect ratios<br>Learning rate {1e-3, 1e-4, 1e-5}<br>Mean squared error optimizer<br>Batch size 12	0.78	0.84

	Adam optimizer + Lookahead<br><br>Max Epochs 50<br><br>Image augmentation (rotation, mirroring, scaling)		
**Segmented model**			
**Fixed size 256x256 images of segmented vessels, keeping stenoses only**	Three frames as input<br><br>Learning rate {1e-3, 1e-4, 1e-5}<br><br>Mean squared error optimizer<br><br>Batch size 12<br><br>Adam optimizer<br><br>Max Epochs 50<br><br>No image augmentation	0.65	0.68
**Fixed size 256x256 images of segmented vessels, enriched with 10000 healthy vessel images**	Three frames as input<br><br>Learning rate {1e-3, 1e-4, 1e-5}<br><br>Mean squared error optimizer<br><br>Batch size 12<br><br>Adam optimizer<br><br>Max Epochs 50<br><br>No image augmentation	0.75	0.83
**Fixed size 256x256 images of segmented vessels, enriched with 10000 healthy vessel images**	Three frames as input<br><br>Learning rate {1e-3, 1e-4, 1e-5}	0.79	0.84

	Mean squared error optimizer<br><br>Batch size 12<br><br>Adam optimizer<br><br>Max Epochs 50<br><br>Image augmentation (rotation, mirroring, scaling)		

Supplementary Table 8. Cath AI classification performance of angiographic projection angle at the image-level in the test dataset (Algorithm 1).

Class	Positive Predictive Value	Sensitivity	F1 Score	Frames	Unique Videos
**RAO Cranial**	0.88	0.93	0.90	37,638	21,936
**AP Cranial**	0.88	0.86	0.87	28,957	17,078
**LAO Cranial**	0.91	0.93	0.92	44,885	28,119
**RAO Straight**	0.91	0.86	0.88	39,563	27,617
**AP**	0.81	0.83	0.82	15,707	9,509
**LAO Straight**	0.94	0.91	0.93	57,028	34,029
**RAO Caudal**	0.84	0.86	0.85	19,552	14,305
**AP Caudal**	0.90	0.88	0.89	22,416	13,278
**LAO Caudal**	0.93	0.96	0.94	31,665	19,988
**LAO Lateral**	0.91	0.88	0.90	1570	751
**RAO Lateral**	0.73	0.66	0.70	485	310
**Other**	0.18	0.08	0.11	159	117
**Overall weighted average**	**0.90**	**0.90**	**0.90**	**299,625**	**110,139**

Results are calculated on the hold-out Test Dataset for Algorithm 1. **Abbreviations:** RAO: Right Anterior Oblique; AP: Antero-posterior; LAO: Left Anterior Oblique.

Supplementary Table 9. CathAI performance for object localization (Algorithm 3).

Classes	Number of object labels in the Test Dataset (N)	Average Precision (%)
**Algorithm 3a: Left and right coronary artery (N = 234 images)**		
**Left coronary artery segments**	**285**	**37.0**
Left main*	48	49.8
Proximal LAD*	70	20.9
Mid LAD*	43	34.8
Distal LAD*	44	40.5
Proximal/mid circumflex*	53	41.0
Distal circumflex*	27	35.8
**Right coronary artery segments**	**353**	**42.8**
Proximal RCA *	81	50.2
Mid RCA *	70	41.8
Distal RCA *	82	29.8
PDA *	72	50.6
PL *	48	41.7
**Other classes**		
Stenosis	93	13.7
Stent/Balloon	16	9.8
Catheter	208	76.1
Guidewire	8	73.2
Sternotomy	78	81.6

Valve	3	100.0
Pacemaker	19	64.9
**Overall Weighted average**	**-**	**48.1**
**Algorithm 3b: Right coronary artery in LAO straight projection (N = 45 images in the Test Dataset)**		
**Right coronary artery segments**	**118**	**54.5%**
Proximal RCA *	29	40.2
Mid RCA *	24	83.2
Distal RCA *	28	55.4
PDA *	22	36.7
PL *	15	57.1
**Other Classes**		
Stenosis	23	26.0
Obstruction	2	0.0
Stent/Balloon	2	0.0
Catheter	45	79.2
Guidewire	8	73.2
Sternotomy	12	92.6
Valve	3	66.7
Pacemaker	9	51.3
**Overall Weighted Average†**	**-**	**58.1**

Abbreviations: LAD: Left Anterior Descending; RCA: Right Coronary Artery; PDA: Posterior Descending Artery; PL: Posterolateral; Mid: Middle; mAP: Mean Average Precision.

*Different coronary artery sub-segments.

†: Average is weighted by the frequency of each class.

Supplementary Table 9: Performance of AI-stenosis (CathAI) versus REPORT-stenosis at the Video and Frame Level (Algorithm 4)

	**Number of REPORT-stenosis labels**	**AUC (95% CI) to discriminate </≥ 70% stenosis severity**	**Mean Absolute difference, % stenosis (continuous) ± SD**	**r* (95% CI)**	**ICC* (95% CI)**	**Sensitivity (95% CI)****	**Specificity (95% CI)** for </≥ 70% stenosis severity**	**PPV (95% CI)** for </≥ 70% stenosis severity**	**NPV (95% CI)** for </≥ 70% stenosis severity**	**MSE* ± SD**
**Video level**	2,766	0.814 (0.797-0.831)	18.8±15.8	0.70 (0.68-0.72)	0.66 (0.54-0.80)	70.2 (66.7-73.5)	72.4 (70.6-74.3)	44.1 (41.2-47.0)	88.7 (87.2-90.0)	602.5±868.9
**Frame level**	14,756	0.757 (0.749-7.645)	19.2±15.1	0.59 0.58-0.60)	0.50 (0.35-0.70)	80.1 (79.1-81.0)	51.7 (50.7-52.6)	52.9 (52.0-53.9)	79.3 (78.4-80.2)	594.97±855.24

Abbreviations: ICC: Intra-class correlation; *r*: Pearson correlation; AUC: Area Under the Receiver Operating Characteristic Curve; PPV: Positive Predictive Value; NPV: Negative Predictive Value; MSE: Mean Squared Error

*Calculation is between AI-stenosis (continuous) and REPORT-stenosis; **The threshold for determining sensitivity, specificity, PPV and NPV for AI-stenosis was 54%.

Supplementary Table 10. Sub-group analysis for AI-stenosis compared to REPORT-stenosis

	Continuous AI-stenosis					Dichotomous AI-stenosis ≥70% stenosed			
	**Number of cases**	**MSE ± SD**	**Absolute difference ± SD**	**r**	**ICC**	**Sensitivity**	**Specificity**	**AUC (95% CI)**	**Diagnostic OR (95% CI)**
**Overall (Artery level)**	**1,734**	565.98±848.32	17.9 ± 15.5	0.74 (0.72-0.76)	0.72 (0.60-0.84)	74.5 (70.0-78.4)	78.1 (76.1-80.1)	**0.862 (0.843-0.880)**	**10.43 (7.94-13.7)**
**Left vs. right coronary artery**									
LCA	980	612.2±884.3	19.0±15.8	0.72 (0.68-0.74)	0.69 (0.54-0.85)	76.1 (71.0-81.3)	72.2 (68.9-75.3)	0.836 (0.809-0.862)	8.5 (5.94-12.18)
RCA	754	496.7±799.2	16.4±15.1	0.77 (0.74-0.80)	0.75 (0.65-0.87)	70.9 (62.7-78.0)	85.6 (83.2-87.9)	0.894 (0.868-0.919)	14.98 (9.77-22.98)
**Age Strata**									
Age <40	70	807.1±1229.4	20.6±19.6	0.53	0.50	45.5	86.7	0.855 (0.754-0.955)	5.42 (1.39-21.12)
Age 40-65	814	545.1±838.0	17.6±15.4	0.75	0.73	76.8	81.2	0.885 (0.86-0.91)	14.32 (9.22-22.23)
Age ≥65	834	553.2±810.7	17.9±15.2	0.74	0.72	74.5	73.8	0.839 (0.811-0.868)	8.24 (5.72-11.85)
**Localization within the coronary artery**									
Proximal segment	892	551.4±742.9	18.6±14.4	0.74	0.72	75.4	187	0.855 (0.828-0.882)	9.65 (6.63-14.03)
Mid segment	401	670.4±965.3	20.2±16.2	0.70	0.62	79.4	97	0.847 (0.807-0.887)	9.6 (5.53-16.66)
Distal segment	441	484.8±929.8	14.5±16.6	0.74	0.77	64.6	65	0.893 (0.861-0.925)	13.1 (7.23-23.74)

**Coronary artery segments**									
Left Main	205	531.3±685.6	18.5±13.8	0.76	0.74	29.6	84.8	0.737 (0.621-0.854)	1.9 (0.48-7.55)
Proximal LAD	282	719.2±1127.1	19.6±18.3	0.65	0.56	79.1	66.3	0.839 (0.792-0.885)	7.44 (4.09-13.53)
Mid LAD	173	786.6±1137.9	20.9±18.8	0.66	0.62	81.3	64.8	0.799 (0.732-0.866)	7.98 (3.54-17.98)
Distal LAD	99	605.3±821.1	19.4±15.1	0.72	0.68	83.3	73.6	0.874 (0.774-0.973)	13.91 (2.83-68.31)
Proximal/MID LCx	191	129.5±357.0	5.7±10.0	0.83	0.96	77.1	65.7	0.802 (0.738-0.867)	6.45 (3.03-13.75)
Distal LCx	30	458.3±633.2	16.5±13.7	0.78	0.74	100.0	93.1	0.0 (0.0-0.0)	inf (nan-inf)
Proximal RCA	214	633.4±822.3	20.6±14.4	0.74	0.67	78.6	86.6	0.908 (0.864-0.952)	23.75 (10.07-56.02)
Mid RCA	228	467.7±864.3	14.4±16.2	0.79	0.76	77.6	76.0	0.879 (0.828-0.929)	10.93 (5.14-23.21)
Distal RCA	121	518.6±1062.8	15.2±17.0	0.74	0.77	72.0	89.6	0.939 (0.898-0.979)	22.11 (7.42-65.86)
Right PDA	109	231.0±495.6	9.3±12.1	0.81	0.90	50.0	91.0	0.867 (0.792-0.943)	10.12 (3.24-31.61)
Right Posterolateral	82	531.3±685.6	18.5±13.8	0.76	0.74	42.9	96.0	0.952 (0.906-0.999)	18.0 (2.72-119.23)
**Abbreviations**: MSE: Mean Squared Error; SD: Standard Deviation; ICC: Intra-class correlation coefficient; AUC: Area Under the Receiver Operating Characteristic Curve; CI: Confidence Interval; OR: Odds-Ratio; LCA: Left Coronary Artery; RCA: Right Coronary Artery; LAD: Left Anterior Descending Artery; LCx: Left Circumflex artery; PDA: Posterior Descending Artery.									

Supplementary Table 11. Sub-group analysis for AI-stenosis compared to QCA-stenosis

	Continuous AI-stenosis					Dichotomous AI-stenosis ≥70% stenosed			
	Number of cases	MSE	Absolute difference	r	ICC	Sensitivity	Specificity	AUC (95% CI)	Diagnostic OR (95% CI)
	**Performance after fine-tuning Algorithm 4**								
**Overall (Frame level)**	**4662**	93.7±197.9	7.4±6.2	0.32	0.48(0.45-0.51)	0.75	0.69	0.774 (0.749-0.799)	6.59 (5.18-8.37)
**Overall (Artery level)**	**357**	72.24±180.24	6.5±5.5	0.24	0.28(0.11-0.42)	0.75	0.80	0.775 (0.594-0.955)	11.79 (3.11-44.69)
	**Left vs. right coronary artery**								
LCA	**162**	66.0±98.8	6.5±4.9	0.24	0.25(0.01-0.44)	0.83	0.77	0.81 (0.609-1.0)	16.98 (1.93-149.26)
RCA	**195**	79.8±245.2	6.6±6.0	0.24	0.3(0.04-0.49)	0.67	0.83	0.739 (0.432-1.0)	9.56 (1.66-54.84)

Supplementary Table 12. Description of concordant/discordant AI-stenosis and REPORT-stenosis in the test dataset.

	**Concordant (%) (N = 1,336 stenoses)**	**Discordant (%) (N = 398 stenoses)**	**p-value***
**Age, y, mean±sd**	62.7±13.2	65.1±12.3	<0.001
**Target artery**			
Right coronary artery	626 (46.9%)	128 (32.2%)	<0.001
Left coronary artery	710 (53.1%)	270 (67.8%)	
**Stenosis localization**			
Proximal RCA	182 (13.6%)	32 (8.0%)	0.008
Mid RCA	174 (13.0%)	54 (13.6%)	0.39
Distal RCA	104 (7.8%)	17 (4.3%)	0.03
Right posterolateral	75 (5.6%)	7 (1.8%)	0.02
Right posterior descending artery	91 (6.8%)	18 (4.5%)	0.17
Left main	159 (11.9%)	46 (11.6%)	0.19
Proximal/Mid LCx	131 (9.8%)	60 (15.1%)	0.54
Distal LCx	28 (2.1%)	2 (0.5%)	0.08
Proximal LAD	198 (14.8%)	84 (21.1%)	0.74
Mid LAD	120 (9.0%)	53 (13.3%)	0.73

Distal LAD	74 (5.5%)	25 (6.3%)	<0.001

Abbreviations: RCA: Right Coronary Artery; LAD: Left Anterior Descending Artery; LCX: Left Circumflex.

*For age, comparisons were done between concordant and discordant stenoses. For stenosis/vessel level data, a mixed effects logistic regression model as used to account for within-subject correlation and for repeated angiograms. Two-sided p-values are presented.

Supplementary References

1. R., P., Manesh *et al.* ACC/AATS/AHA/ASE/ASNC/SCAI/SCCT/STS 2017 Appropriate Use Criteria for Coronary Revascularization in Patients With Stable Ischemic Heart Disease: A Report of the American College of Cardiology Appropriate Use Criteria Task Force, American Association for Thoracic Surgery, American Heart Association, American Society of Echocardiography, American Society of Nuclear Cardiology, Society for Cardiovascular Angiography and Interventions, Society of Cardiovascular Computed Tomography, and Society of Thoracic Surgeons. *J Am Coll Cardiol* 69, 2212–2241 (2017).

2. David, K. *et al.* Comparative Validation of Quantitative Coronary Angiography Systems. *Circulation* 91, 2174–2183 (1995).

3. Kussmaul, W. G., Popp, R. L. & John, N. Accuracy and reproducibility of visual coronary stenosis estimates using information from multiple observers. *Clin Cardiol* 15, 154–162 (1992).

4. Leape, L. L. *et al.* Effect of variability in the interpretation of coronary angiograms on the appropriateness of use of coronary revascularization procedures. *American Heart Journal* 139, 106–113 (2000).

5. Georgios, S. *et al.* The SYNTAX Score: an angiographic tool grading the complexity of coronary artery disease. *EuroIntervention* 1, 219–227 (2005).
