# Supplementary material for: CathAI: fully automated coronary angiography interpretation and stenosis estimation
Source: NPJ Digit Med. 2023 Aug 11;6:142. doi: 10.1038/s41746-023-00880-1 (PMC10421915; doi:10.1038/s41746-023-00880-1)
Supplement: Supplementary file 2 — Reporting Summary [file 41746_2023_880_MOESM2_ESM.pdf]

nature portfolio

Corresponding author(s): Geoffrey H Tison

Last updated by author(s): 7/9/2023

# Reporting Summary

Nature Portfolio wishes to improve the reproducibility of the work that we publish. This form provides structure for consistency and transparency in reporting. For further information on Nature Portfolio policies, see our Editorial Policies and the Editorial Policy Checklist.

## Statistics

For all statistical analyses, confirm that the following items are present in the figure legend, table legend, main text, or Methods section.

| n/a | Confirmed | |
|---|---|---|
| ☐ | ☒ | The exact sample size (*n*) for each experimental group/condition, given as a discrete number and unit of measurement |
| ☐ | ☒ | A statement on whether measurements were taken from distinct samples or whether the same sample was measured repeatedly |
| ☐ | ☒ | The statistical test(s) used AND whether they are one- or two-sided<br>*Only common tests should be described solely by name; describe more complex techniques in the Methods section.* |
| ☐ | ☒ | A description of all covariates tested |
| ☒ | ☐ | A description of any assumptions or corrections, such as tests of normality and adjustment for multiple comparisons |
| ☐ | ☒ | A full description of the statistical parameters including central tendency (e.g. means) or other basic estimates (e.g. regression coefficient) AND variation (e.g. standard deviation) or associated estimates of uncertainty (e.g. confidence intervals) |
| ☐ | ☒ | For null hypothesis testing, the test statistic (e.g. *F*, *t*, *r*) with confidence intervals, effect sizes, degrees of freedom and *P* value noted<br>*Give P values as exact values whenever suitable.* |
| ☒ | ☐ | For Bayesian analysis, information on the choice of priors and Markov chain Monte Carlo settings |
| ☒ | ☐ | For hierarchical and complex designs, identification of the appropriate level for tests and full reporting of outcomes |
| ☐ | ☒ | Estimates of effect sizes (e.g. Cohen's *d*, Pearson's *r*), indicating how they were calculated |

*Our web collection on statistics for biologists contains articles on many of the points above.*

## Software and code

Policy information about availability of computer code

| | |
|---|---|
| Data collection | Data were obtained during the course of clinical care from UCSF and the University of Ottawa Heart Institute as well as from the Montreal Heart Institute Angiographic Core Lab. |
| Data analysis | All analyses were performed using Python 2.7. Neural networks were trained using Keras v.2.24 and TensorFlow v.1.12.  Custom code was developed for all analyses and algorithms, in some cases based on open source architectures as described in the Methods. |

For manuscripts utilizing custom algorithms or software that are central to the research but not yet described in published literature, software must be made available to editors and reviewers. We strongly encourage code deposition in a community repository (e.g. GitHub). See the Nature Portfolio guidelines for submitting code & software for further information.

## Data

Policy information about availability of data

All manuscripts must include a data availability statement. This statement should provide the following information, where applicable:

- Accession codes, unique identifiers, or web links for publicly available datasets
- A description of any restrictions on data availability
- For clinical datasets or third party data, please ensure that the statement adheres to our policy

The data used in this study are derived from clinical care and thus are not made publicly available due to data privacy concerns. Reasonable requests for collaboration using the data can be made from the authors, as feasible and permitted by the Regents of the University of California.

## Research involving human participants, their data, or biological material

Policy information about studies with human participants or human data. See also policy information about sex, gender (identity/presentation), and sexual orientation and race, ethnicity and racism.

| | |
|---|---|
| Reporting on sex and gender | We relied on self reporting of sex as available in the angiogram meta-data. This was used to perform sex-stratified analyses of the main results of CathAI Algorithm 4 in Table 2. |
| Reporting on race, ethnicity, or other socially relevant groupings | Reliable information on race/ethnicity were not readily available in the angiograic meta-data or reporting systems, hence this was not reported in this study. |
| Population characteristics | The Full Dataset consisted of 13,843 complete angiogram studies (195,195 total angiographic videos) from 11,972 patients aged ≥18 years from the University of California, San Francisco (UCSF), between April 1, 2008 and December 31, 2019. Mean age was 63.5 ±13.7 years in the Full UCSF Dataset. |
| Recruitment | No specific recruitment was performed for this study, as data were obtained during the course of clinical care as described for each dataset. |
| Ethics oversight | IRB approval was obtained from the UCSF and UOHI IRB review boards. |

Note that full information on the approval of the study protocol must also be provided in the manuscript.

# Field-specific reporting

Please select the one below that is the best fit for your research. If you are not sure, read the appropriate sections before making your selection.

☒ Life sciences ☐ Behavioural & social sciences ☐ Ecological, evolutionary & environmental sciences

For a reference copy of the document with all sections, see nature.com/documents/nr-reporting-summary-flat.pdf

# Life sciences study design

All studies must disclose on these points even when the disclosure is negative.

| | |
|---|---|
| Sample size | For the UCSF training dataset, all angiograms obtained at UCSF during they study period were used. This maximized the available data for the training dataset for optimal algorithm development. |
| Data exclusions | No data were excluded from the UCSF dataset. For the UOHI dataset, angiograms were randomly sampled within the study period to generate an external validation dataset. |
| Replication | Results were validated internally at UCSF, then also at the UOHI. |
| Randomization | There were no experimental groups in our study. |
| Blinding | There was no intervention, so blinding is not relevant in our study. |

# Reporting for specific materials, systems and methods

We require information from authors about some types of materials, experimental systems and methods used in many studies. Here, indicate whether each material, system or method listed is relevant to your study. If you are not sure if a list item applies to your research, read the appropriate section before selecting a response.

### Materials & experimental systems

| n/a | Involved in the study | |
|---|---|---|
| ☒ | ☐ | Antibodies |
| ☒ | ☐ | Eukaryotic cell lines |
| ☒ | ☐ | Palaeontology and archaeology |
| ☒ | ☐ | Animals and other organisms |
| ☐ | ☒ | Clinical data |
| ☒ | ☐ | Dual use research of concern |
| ☒ | ☐ | Plants |

### Methods

| n/a | Involved in the study | |
|---|---|---|
| ☒ | ☐ | ChIP-seq |
| ☒ | ☐ | Flow cytometry |
| ☒ | ☐ | MRI-based neuroimaging |

# Clinical data

Policy information about clinical studies

All manuscripts should comply with the ICMJE guidelines for publication of clinical research and a completed CONSORT checklist must be included with all submissions.

| | |
|---|---|
| Clinical trial registration | This was not a clinical trial. |
| Study protocol | There was no protocol for this development and validation study. |
| Data collection | The UCSF Dataset consisted of 13,843 complete angiogram studies (195,195 total angiographic videos) from 11,972 patients aged ≥18 years from the University of California, San Francisco (UCSF), between April 1, 2008 and December 31, 2019. We randomly sampled coronary angiogram videos in patients ≥ 18 years old, from the University of Ottawa Heart Institute (UOHI) obtained between July 1st 2020 and October 31st 2020. |
| Outcomes | There were no specific outcomes for this study. Severe angiographic stenosis was defined at ≥70% as per clinical guidelines. |
